# Functional Relationships between L1CAM, LC3, ATG12, and Aβ

**DOI:** 10.3390/ijms251910829

**Published:** 2024-10-09

**Authors:** Gabriele Loers, Ute Bork, Melitta Schachner

**Affiliations:** 1 Zentrum für Molekulare Neurobiologie, Universitätsklinikum Hamburg-Eppendorf, Martinistrasse 52, 20246 Hamburg, Germany; 2 Department of Cell Biology and Neuroscience, Keck Center for Collaborative Neuroscience, Rutgers University, 604 Allison Road, Piscataway, NJ 08854, USA

**Keywords:** L1CAM, LC3, LIR, Aβ, autophagy, Alzheimer’s disease

## Abstract

Abnormal protein accumulations in the brain are linked to aging and the pathogenesis of dementia of various types, including Alzheimer’s disease. These accumulations can be reduced by cell indigenous mechanisms. Among these is autophagy, whereby proteins are transferred to lysosomes for degradation. Autophagic dysfunction hampers the elimination of pathogenic protein aggregations that contribute to cell death. We had observed that the adhesion molecule L1 interacts with microtubule-associated protein 1 light-chain 3 (LC3), which is needed for autophagy substrate selection. L1 increases cell survival in an LC3-dependent manner via its extracellular LC3 interacting region (LIR). L1 also interacts with Aβ and reduces the Aβ plaque load in an AD model mouse. Based on these results, we investigated whether L1 could contribute to autophagy of aggregated Aβ and its clearance. We here show that L1 interacts with autophagy-related protein 12 (ATG12) via its LIR domain, whereas interaction with ubiquitin-binding protein p62/SQSTM1 does not depend on LIR. Aβ, bound to L1, is carried to the autophagosome leading to Aβ elimination. Showing that the mitophagy-related L1-70 fragment is ubiquitinated, we expect that the p62/SQSTM1 pathway also contributes to Aβ elimination. We propose that enhancing L1 functions may contribute to therapy in humans.

## 1. Introduction

Neural cell adhesion molecules are glycoproteins that mediate interactions of nervous system cells with each other and with the extracellular matrix. As transmembrane molecules, they act as receptors at the cell surface. Their interactions can be homophilic or heterophilic with different binding partners. When stimulated at the cell surface, they induce intracellular signaling cascades, modulate transcription, and change cell adhesion, migration and survival [1,2]. One important cell adhesion molecule is L1CAM, L1 in short, which contributes to neuronal cell differentiation, axon fasciculation, axon guidance, migration of neural cells, myelination, synapse formation, and synaptic plasticity [2,3,4]. L1 is also expressed by immune cells and tumors, including melanomas, neuroblastomas, glioblastomas, and other tumor cell types, such as, for instance, renal, colon, endometrial, and ovarian carcinomas [5,6,7,8,9,10]. In tumors, L1 functions are linked to cell motility, malignancy, metastasis, and, unexpectedly, drug resistance. Thus, L1 expression can be used as a marker for poor prognosis of afflicted patients [8,9,11,12]. The molecular structure of L1 consists at the N-terminal extracellular part of six immunoglobulin-like domains and five fibronectin type III homologous repeats, which are followed by one transmembrane domain and a short intracellular domain at the C-terminus [2,8]. Mutations in the L1 gene can lead to the so-called L1 syndrome, as described in humans and mice [8,13,14,15,16]. The L1 syndrome varies in severity depending on the genetic background and is characterized by X-linked hydrocephalus, stenosis of the aqueduct of Sylvius, corpus callosum agenesis, congenital aganglionosis, aphasia, adducted thumbs, spastic paraplegia type 1, and mental retardation [13,14,15,17,18,19,20]. Such abnormalities are also seen, for instance, in the fetal alcohol syndrome, Alzheimer’s disease (AD), schizophrenia, and autism [21,22,23,24,25,26,27].

The physiological functions of L1 are closely linked to L1’s cleavage by different proteases, including metallo- and serine proteases, which contribute to motility of L1 expressing cells. These proteases not only generate soluble extracellular but also intracellular fragments of L1 [2,8,9,28,29,30,31,32,33,34,35,36]. Of particular interest is a 70 kDa transmembrane fragment of L1 (L1-70) generated by myelin basic protein which induces signal transduction [28,29]. Remarkably, L1-70 is imported into mitochondria, where it increases ATP production and contributes to mitochondrial homeostasis [29,30,31,37,38,39,40]. Regarding AD, it is remarkable that L1-70 is upregulated in a mouse model of AD where parabiosis of the AD mouse with a young adult wild-type mouse reduces Aβ deposits and attenuates the disease symptoms [41]. Also, abnormally high levels of soluble L1 fragments are observed in the cerebrospinal fluid of patients with AD symptoms and other dementia-like behavior [24] as well as in the serum and ascites of ovarian carcinoma patients [42,43].

Dysregulation of proteostasis-associated pathways has been identified as a risk factor for neurodegenerative diseases and age-dependent neuronal impairments. These impairments are strongly based on dysregulation of autophagy and of mitochondrial functions, also found in AD [44,45,46,47,48,49]. Proteolysis and mitophagy regulate neuroprotection by maintaining healthy mitochondria through macroautophagy [50]. Aβ and hyper-phosphorylated tau are considered to be the most important players in AD. They contribute to the development of pathological conditions by (i) reducing mitochondrial trafficking, (ii) blocking of bi-directional mitochondrial transport at or close to the synapse, (iii) enhancing mitochondrial fission and swelling of mitochondria by increasing the influx of water through mitochondrial permeability transition openings, and (iv) reducing ATP production by blocking the mitochondrial activities of complex I and complex IV [47,51]. Of note, L1-deficient mice manifest impaired complex I activity, impaired mitochondrial membrane potential, and reduced mitochondrial trafficking velocity. Furthermore, regulation of complex I activity by L1 depends on the import of L1-70 into mitochondria [30].

We have reported that L1 binds to Aβ and that overexpression of L1 or upregulation of L1 fragments in mouse models for Alzheimer’s disease reduce Aβ1-42 levels and Aβ plaques, activate macrophages, and ameliorate loss of inhibitory perisomatic synapses on cornu ammonis (CA)1 and CA3 pyramidal cells [26,41]. We also showed that L1 increases mitochondrial activity and neuronal cell survival via its interaction with microtubule-associated protein 1 light-chain 3 (LC3) [52], which is a key regulator of endocytosis, phagocytosis, secretory autophagy, and autophagy [53]. Binding of proteins to LC3 and autophagy-related proteins (ATGs) is predominantly mediated by the LC3-interacting region (LIR) [54,55,56]. L1 contains the LIR motif _952_LSYHPV_957_ in its fourth fibronectin type III homologous repeat and interacts with LC3 via this motif [52]. In vertebrates, proteins with a LIR motif regulate removal of damaged mitochondria by mitophagy and of protein aggregates by autophagy [54,55,56,57,58]. Generation of protein aggregates and damaged mitochondrial homeostasis are not only pathological hallmarks of AD but also of other neurodegenerative diseases [59,60,61]. Thus, proper functioning of selective autophagy and mitophagy are essential for sustaining healthy cells and healthy individuals [54,59,62]. On the basis of these findings, we now focused on investigating the effects of L1 on mitophagy and autophagy as well as on Aβ clearance. Towards this aim, we searched for further interaction partners of L1 that are involved in regulating autophagy and investigated Aβ toxicity and autophagic flow. We found that L1 interacts with ATG12 in an LIR-dependent manner, and that L1 is in close proximity to p62/SQSTM1. Amelioration of Aβ-induced toxicity by triggering of L1 in cultured primary neurons depends on L1’s LIR domain. In addition, triggering of L1’s functions in cultured neurons with the small molecule L1 agonistic mimetic duloxetine enhances the interaction of L1 with LC3, ATG12, and p62. Duloxetine-treatment also leads to increased co-localization of Aβ with L1 and of Aβ with the autophagy markers ATG12 and p62 and reduces overall Aβ levels in neuroblastoma cells. Results show that L1 enhances autophagy and is therefore important to maintain healthy cells.

## 2. Results

### 2.1. L1 Binds to LC3, ATG12, and Aβ

**Figure 1:**
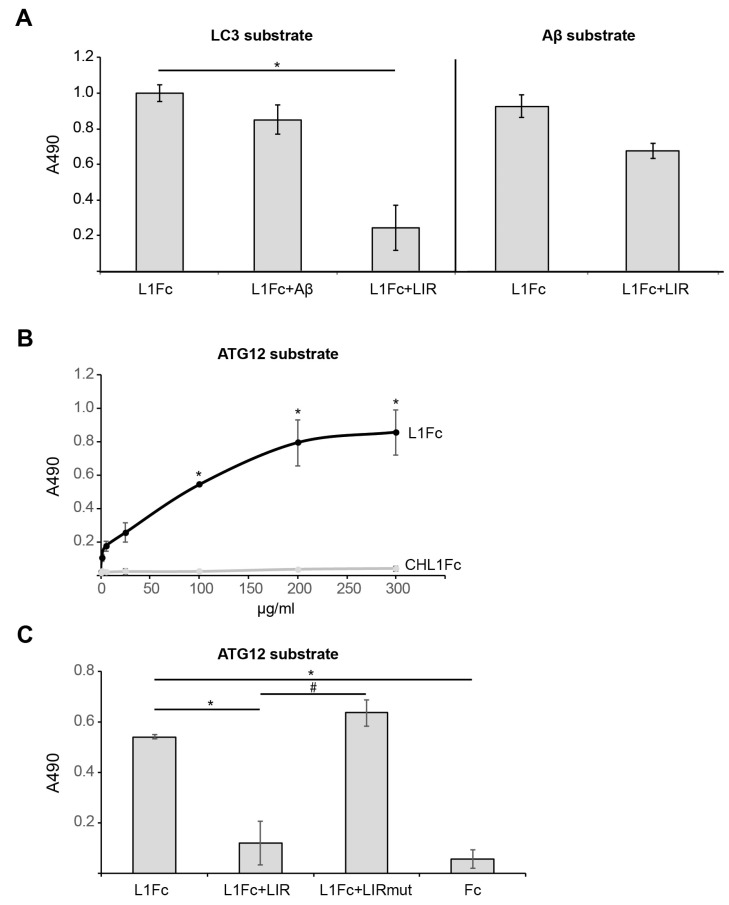
**L1 binds to LC3 and ATG12 via its LIR domain, whereas binding to Aβ is not mediated by the LIR domain.** (**A**) Substrate-coated recombinant LC3B or Aβ1-42 was incubated with L1Fc alone or with L1Fc together with Aβ1-42 or the LIR peptide. (**B**,**C**) Substrate-coated recombinant ATG12 was incubated with different concentrations of L1Fc and CHL1Fc (**B**) or L1Fc in the presence and absence of the LIR peptide, the mutated LIR peptide, and Fc as control (**C**). Via ELISA, binding was determined using horseradish peroxidase-conjugated anti-Fc antibody. Mean values ± SD from three independent experiments carried out in triplicates are shown. * *p* < 0.05 relative to L1Fc (**A**,**C**) or CHL1Fc (**B**), # *p* < 0.05 relative to L1Fc+LIR, Mann–Whitney U-test.

To reiterate, we showed that L1 interacts with Aβ via its extracellular domain [26], and that L1-70 interacts with LC3B via its LIR motif [52]. In addition, L1 reduces Alzheimer’s disease pathology in AD model mice and protects neurons against oxidative stress in vitro and in vivo [26,29,41,63]. Thus, we here investigated the interaction of L1 with autophagy proteins and the influence of L1 on autophagy and Aβ toxicity and clearance. Autophagy is a process that is primarily regulated by ATG family members. LC3B, ATG16L1, AGT5, and ATG12 are essential for autophagy as they allow membrane expansion and fusion of extending membranes of the phagophore so they can combine in a vesicle, the autophagosome [64,65,66]. The autophagy receptor p62/SQSTM1, from now on abbreviated p62, delivers aggregated and ubiquitinated proteins to the proteasome for degradation. It shuttles between the nucleus and cytoplasm, binds with ubiquitinated cargoes, and enables quality control of nuclear and cytosolic proteins [67]. Protein aggregates of Aβ and Tau, which are found in AD, bind to p62 and are not incorporated into LC3-positive autophagosomes [68]. Here, we investigated if L1 not only interacts with LC3 but also with other ATG family member proteins or p62 and asked how L1 may influence autophagy and Aβ aggregate detoxification. First, we determined via ELISA if binding of L1 to Aβ is altered in the presence of a 23-amino-acid-long peptide containing L1’s LIR motif or if binding of L1 to LC3 is altered in the presence of the Aβ1-42 peptide. Recombinant LC3B and Aβ were used as a substrate-coat and incubated with the extracellular domain of L1 fused to Fc from human IgG (L1Fc), in the presence and absence of a peptide containing the LIR motif of L1 or Aβ (Figure 1A). Results confirm our previous observation that the extracellular domain of L1 binds to LC3 and Aβ. Binding of L1Fc to LC3B was reduced in the presence of the LIR peptide, whereas binding between L1 and LC3B was not altered in the presence of Aβ. In addition, binding of L1Fc to Aβ was not reduced in the presence of the LIR peptide (Figure 1A). These results suggest that L1 can bind to LC3B and Aβ simultaneously, and, importantly, that different sites in L1 mediate binding to LC3 and Aβ.

Next, we investigated if L1, via its LIR domain, also binds to ATG12. We found that L1 binds to ATG12 concentration dependently, while no binding of CHL1, the close homolog of L1, to ATG12 was observed (Figure 1B). Binding of L1Fc to ATG12 was reduced by the LIR peptide but not by the mutated LIR peptide (Figure 1C), showing that binding of L1 to ATG12 depends on the LIR motif in L1’s extracellular domain.

To determine if L1, LC3, and Aβ interact in a cellular context and reside in a complex in live cells, co-immunostaining and proximity ligation assay were performed. B103 neuroblastoma cells expressing L1 and APP carrying the Swedish mutation, which leads to Aβ aggregates, were used for achieving this aim. Cells used for immunostaining were treated with solvent control, autophagy inhibitor 3-methyladenine (3-MA), or autophagy inducer rapamycin to elucidate if inducing or inhibiting autophagy changes the levels of co-localization of L1 with Aβ and LC3. Control and 3-MA-treated B103 cells showed co-localization of L1, LC3, and Aβ, while cells treated with rapamycin did not show this (Supplementary Figure S1). In addition, cells were treated for 4 h with function-triggering L1 monoclonal antibody 557 before immunostaining to determine if stimulation of L1 functions at the cell surface alters the co-localization of Aβ and L1 with LC3. Images verify co-localization of L1 with Aβ and LC3, and protein levels of control and 557-treated cells were similar, suggesting that acute stimulation of L1 is not required to trigger these interactions (Supplementary Figure S1).

To examine more precisely the level of co-localization of L1 and LC3 and L1 as well as Aβ, Pearson’s correlation coefficient and Manders’ co-localization coefficients were calculated from control- and 557 antibody-treated cells. The Pearson’s correlation coefficient revealed a moderate co-localization of L1 and Aβ and a strong co-localization of L1 and LC3 in control-treated cells (Supplementary Figure S2). Co-localization coefficients M1 and M2 were low in the case of L1 and Aβ and indicated an approximately 20% overlap, while M1 and M2 were approximately 50% for L1 and LC3, indicating a 50% overlap of L1 and LC3 immunostainings. Interestingly, treatment of cells with function-triggering L1 antibody 557 did not alter the levels of co-localization compared to vehicle-stimulated control cells (Supplementary Figure S2).

### 2.2. Interactions of L1 with LC3, p62, and Aβ Are Enhanced under Oxidative Stress

**Figure 2:**
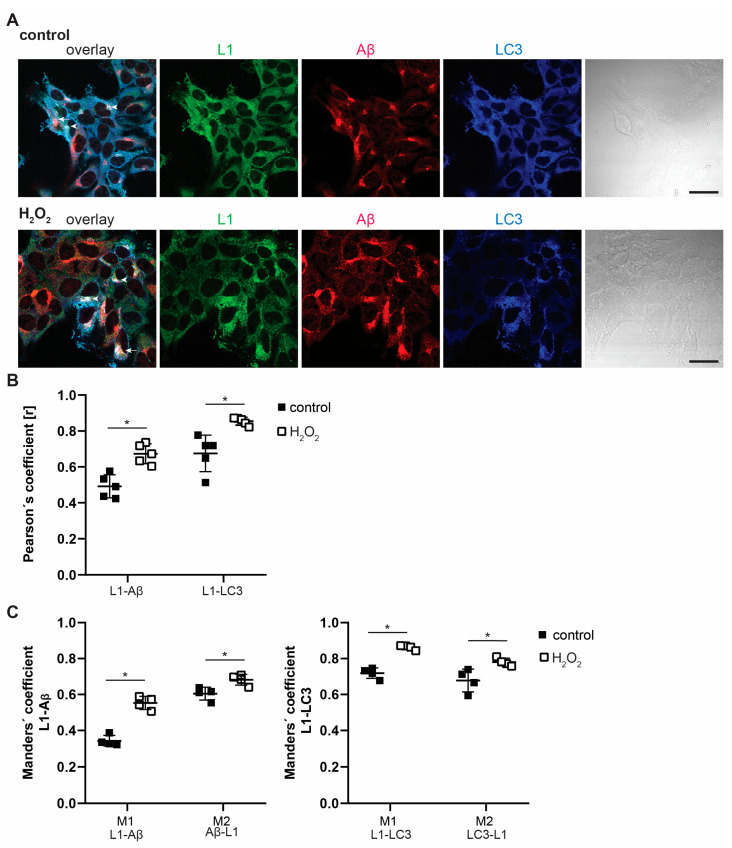
**Hydrogen peroxide treatment enhances co-localization of L1 with Aβ and LC3.** B103 cells were cultured for 48 h, treated with control (H_2_O) and 20 µg H_2_O_2_ for 24 h, fixed, and stained with antibodies against L1, Aβ, and LC3. (**A**) Representative images of cells from 4 independent experiments are shown. Arrows point to yellow dots that indicate co-localization of L1 and Aβ; arrowheads point to white dots showing co-staining of L1, Aβ, and LC3. Scale bars: 20 µm. (**B**,**C**) Co-localization analyses of L1 (green), Aβ (red), and LC3 (blue) was performed and the Pearson’s correlation coefficient and the Manders’ overlap coefficients were determined from four independent cultures. M1: proportion of L1 (green) overlapping with Aβ (red) or LC3 (blue) over the total intensity; M2: proportion of Aβ (red) or LC3 (blue) overlapping with L1 (green) over the total intensity. Average values and means with SD from four and five independent experiments are presented. Data were analyzed with one-way ANOVA with Bonferroni’s multiple comparison test, * *p* < 0.05, relative to control treatment.

Since L1 is neuroprotective and saves cells from oxidative stress [2,16], cells were stressed with hydrogen peroxide (H_2_O_2_) and co-immunostaining of stressed and non-stressed cells was performed (Figure 2). Pearson’s correlation coefficients were enhanced in H_2_O_2_-treated cells compared to solvent-treated control cells, indicating a high level of co-localization of L1 and Aβ as well as of L1 and LC3. Of note, Manders’ co-localization coefficients were also higher in H_2_O_2_-treated cells, indicating a 50–60% overlap of L1 and Aβ immunostaining and an 80–90% overlap of L1 and LC3 immunostaining (Figure 2). These findings confirm that L1 interacts with Aβ in cells and suggest that oxidative stress, which damages mitochondria and cells and induces autophagy and cell death, enhances co-localization and interaction of L1 with Aβ as well as with LC3.

**Figure 3:**
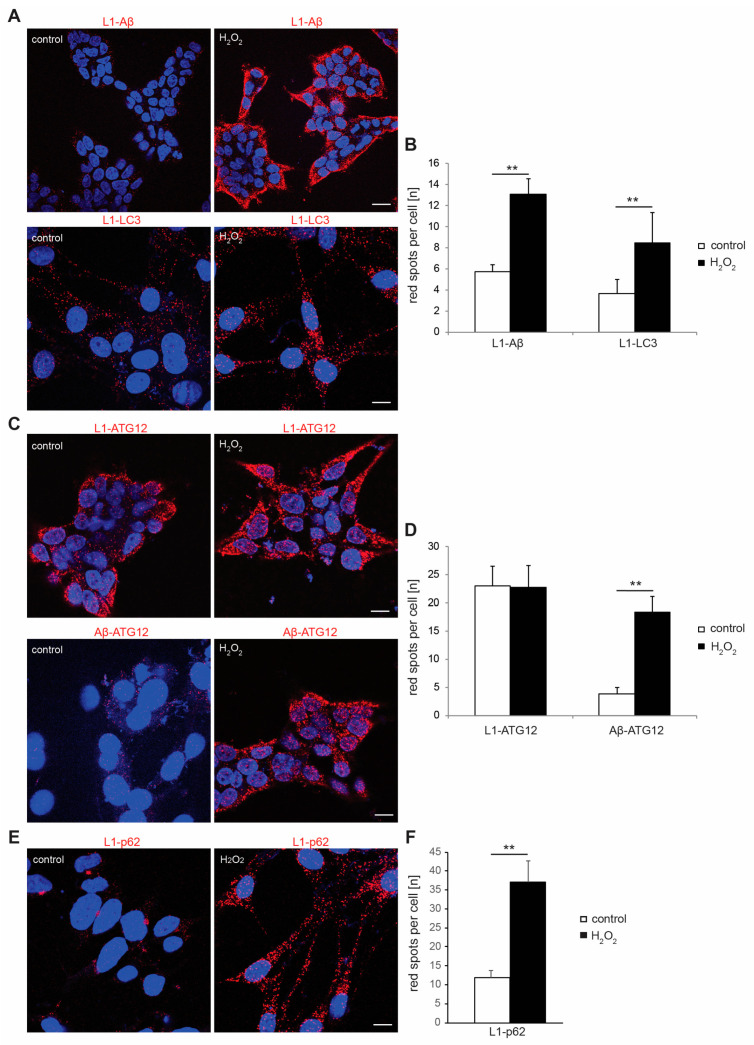
**Interactions of L1 with Aβ, L1 with LC3, L1 with p62, and Aβ with ATG12 are enhanced in B103 cells after induction of oxidative stress.** B103 cells were cultured for 48 h, treated with H_2_O (control) or 20 µM H_2_O_2_ for 24 h, fixed, and subjected to proximity ligation assay with mouse L1 antibody C-2 and rabbit Aβ antibody, rabbit LC3 antibody, goat ATG12 antibody, or rabbit p62 antibody. Nuclei were stained with DAPI (blue). (**A**,**C**,**E**) Representative images are shown and red spots indicate close interaction of L1 with Aβ, L1 with LC3 (**A**), L1 with ATG12, Aβ with ATG12 (**C**), and L1 with p62 (**E**). Scale bars: 20 µm. (**B**,**D**,**F**) Mean values + SD are shown for the average numbers of L1/Aβ- and L1/LC3-positive red spots per cell (**B**), L1/ATG12- and Aβ/ATG12-positive red spots per cell (**D**), and L1/p62-positive red spots per cell (**F**) from three independent experiments. Data were analyzed with one-way ANOVA with Bonferroni’s multiple comparison test, ** *p* < 0.01 relative to control treatment.

To verify that L1 directly interacts with Aβ not only in biochemical assays but also in live cells and to evaluate if induction of oxidative stress alters this interaction, the proximity ligation assay was performed. In this assay, a signal is only seen when proteins are in close proximity and less than 40 nm apart from each other, suggesting a direct interaction [37,52]. Following the observations that L1 directly interacts with LC3 [52], we now tested B103 and HEK293 cells which were stained with antibodies against L1 and LC3 as positive control. In addition, antibodies against L1 and ATG12 as well as L1 and p62 were used to determine whether L1 can also interact with other LIR domain-interacting proteins of the autophagy-related protein family or proteins linking the ubiquitin–proteasome system and autophagy pathway in cells. B103 cells were treated with H_2_O (solvent control) or H_2_O_2_ and stained with antibodies against L1 and Aβ, L1 and ATG12, L1 and p62, or Aβ and ATG12 (Figure 3A–F and Supplementary Figure S3A). Red spots indicating close proximity of L1 with Aβ, LC3, ATG12, and p62 were detected in L1 expressing cells, and the numbers of red spots indicating the interaction of L1 with Aβ, of L1 with LC3, and of L1 with p62 were enhanced 2-fold (L1 with LC3 and L1 with Aβ) or 3-fold (L1 with p62) when cells had been treated with H_2_O_2_, whereas the number of red spots indicating the interaction of L1 and ATG12 were similar between control cells and cells treated with H_2_O_2_ (Figure 3B,D and Supplementary Figure S3B). Interestingly, Aβ was also in close proximity with ATG12, and the number of red spots in cells treated with H_2_O_2_ was 4-fold higher compared to controls (Figure 3D). That the staining was specific was verified with L1-negative HEK293 cells, which were also incubated with antibodies against L1 and Aβ, L1 and ATG12, and L1 and p62. L1-negative HEK293 cells did not show red spots for L1 and Aβ, L1 and ATG12, or L1 and p62 antibodies, proving that results were specific (Supplementary Figure S3). L1 expression of transfected HEK293 cells was tested via immunostaining and Western blot analysis. Images reveal L1 in cell lysates of cells transfected with the L1 plasmid. For control, no L1 signal is seen in lysates of cells transfected with the empty plasmid (Supplementary Figure S4).

**Figure 4:**
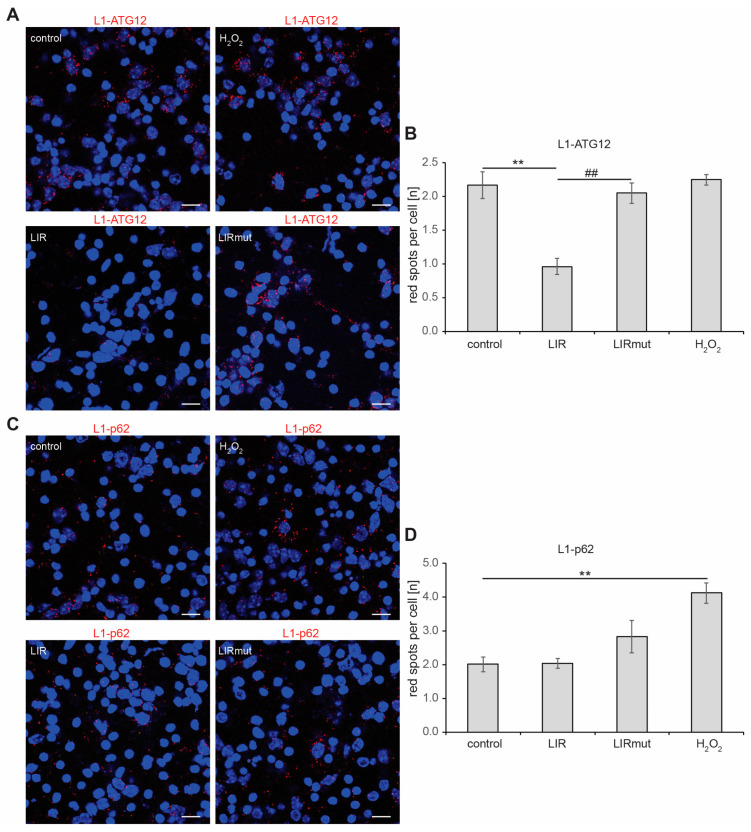
**Interactions of L1 with ATG12, but not of L1 with p62, depend on the LIR motif of L1.** Cortical neurons were allowed to settle down, treated with H_2_O (control), cell-penetrating L1-LIR peptide or mutated LIR peptide, cultured overnight, and treated with H_2_O or 20 µM H_2_O_2_ for 24 h. After fixation, proximity ligation assay with mouse L1 antibody C-2, goat ATG12 antibody, and rabbit p62 antibody was performed. Nuclei were stained with DAPI (blue). (**A**,**C**) Representative images are shown, and red spots indicate close interaction of L1 with ATG12 (**A**) and L1 with p62 (**C**). Scale bars: 20 µm. (**B**,**D**) Mean values ± SD are shown for the average numbers of L1/ATG12-positive (**B**) and L1/p62-positive (**D**) red spots per cell from three independent experiments. Data were analyzed with one-way ANOVA with Bonferroni’s multiple comparison test, ** *p* < 0.01 relative to control treatment, ## *p* < 0.01 relative to LIR treatment.

Similar results were obtained when primary cortical neurons were used for proximity ligation. L1 was seen in close proximity to ATG12 and p62, and treatment with hydrogen peroxide enhanced the interaction of L1 with p62 but not with ATG12 (Figure 4). Furthermore, when cells were treated with a cell-penetrating peptide containing L1’s LIR domain, the interaction of L1 with ATG12 was strongly reduced, whereas the interaction with p62 was not changed (Figure 4). A control peptide with the mutated LIR domain did not affect the interaction of L1 with ATG12, thereby verifying that L1 interacts with ATG12 via its LIR domain.

**Figure 5:**
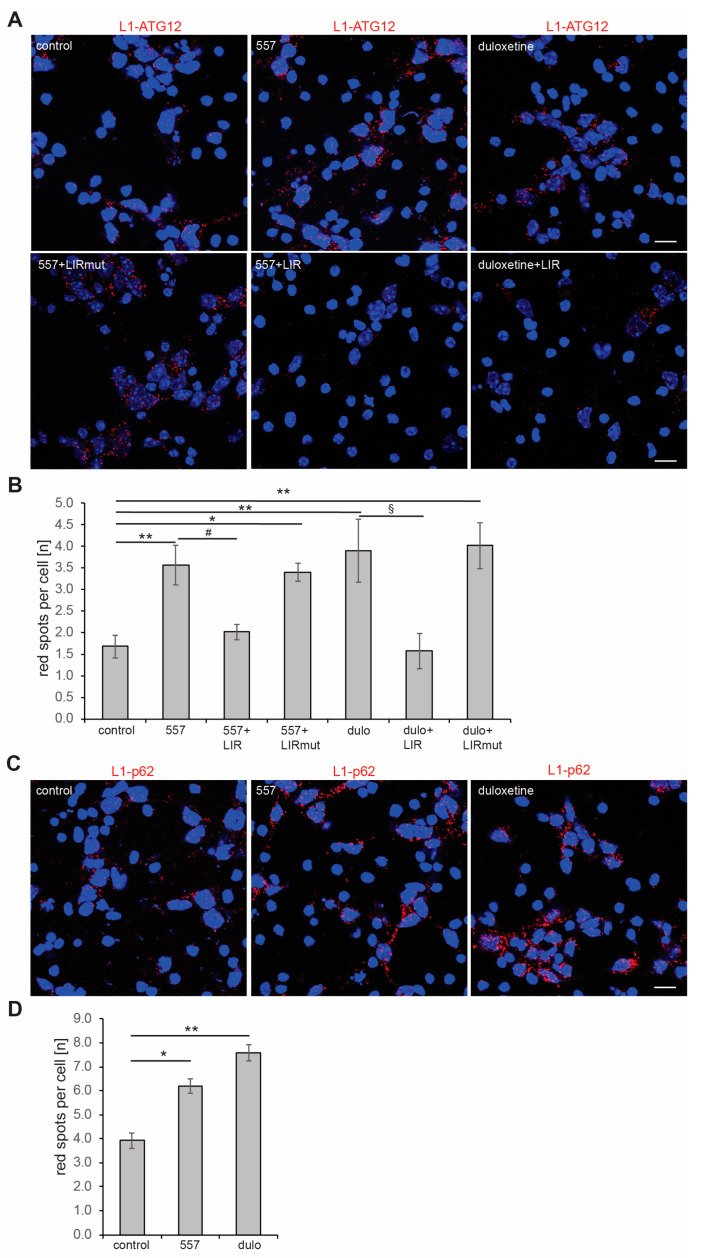
**Triggering L1 functions enhances the interactions of L1 with ATG12 and p62.** Cortical neurons (**A**–**D**) were allowed to settle down, treated with 0.001% DMSO (solvent control), cell-penetrating L1-LIR peptide (LIR), or mutated LIR peptide (LIRmut) for 30 min and then with PBS, function-triggering L1 antibody 557, or function-triggering L1 agonistic mimetic duloxetine (dulo) for 24 h. Thereafter, cells were fixed and proximity ligation assay with mouse L1 antibody C-2, goat ATG12 antibody, or rabbit p62 antibody was performed. Nuclei were stained with DAPI (blue). (**A**,**C**) Representative images are shown, and red spots indicate close interaction of L1 with ATG12 (**A**) and L1 with p62 (**C**). Scale bars: 20 µm. Mean values ± SD are shown for the average numbers of L1/ATG12-positive (**B**) and L1/p62-positive (**D**) red spots per cell from three independent experiments. Data were analyzed with one-way ANOVA with Bonferroni’s multiple comparison test, * *p* < 0.05 and ** *p* < 0.01 relative to control treatment, # *p* < 0.05 relative to 557 antibody treatment, ^§^ *p* < 0.05 relative to duloxetine treatment.

When cortical neurons and B103 cells were treated with function-triggering monoclonal L1 antibody 557 or the L1 agonistic mimetic duloxetine, the numbers of red dots indicating the interactions of L1 with ATG12, L1 with p62, L1 with LC3, and L1 with Aβ were enhanced (Figure 5; Supplementary Figures S5 and S6). Furthermore, the LIR peptide, but not the mutated LIR peptide, reduced the numbers of red dots indicative of L1-ATG12 interactions, and values of cells treated with the LIR peptide are comparable to those of non-stimulated control cells (Figure 5 and Supplementary Figure S5). These results show that the interaction of L1 with ATG12 in 557 antibody- and duloxetine-stimulated cells is specific and depends on L1’s LIR domain. Furthermore, the results strengthen the notion that the LIR motif in the extracellular domain of L1 is crucial for L1’s interaction with ATG12. These results also show that interactions of L1 with ATG12 and L1 with p62 are enhanced when L1 is triggered.

**Figure 6:**
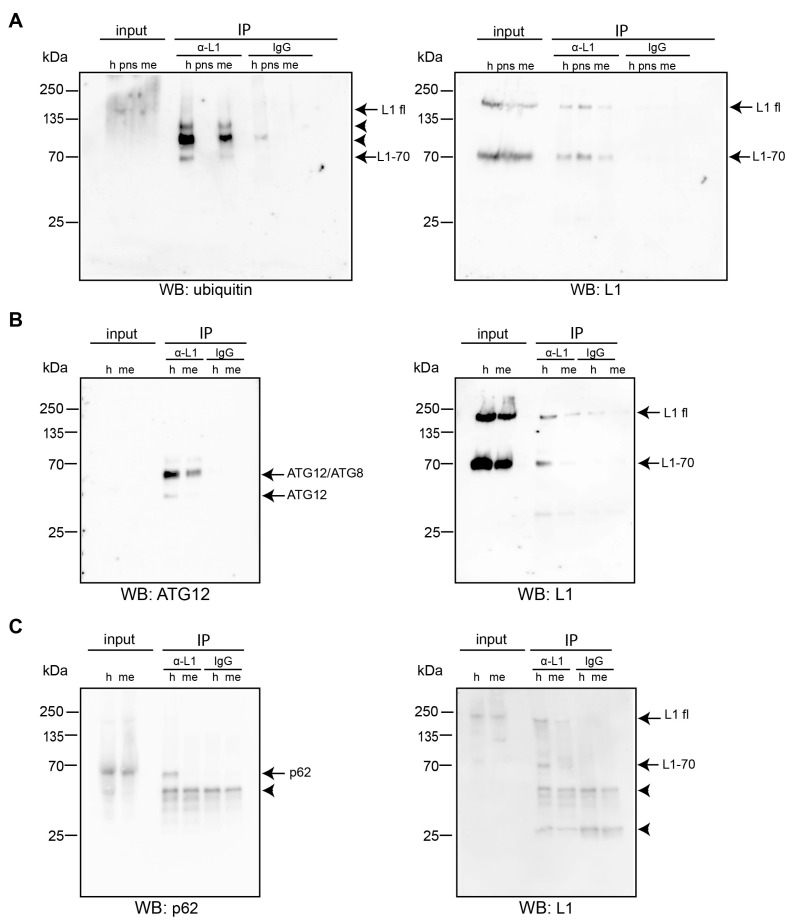
**L1 is ubiquitinated and co-immunoprecipitates with ATG12 and p62.** Brain homogenate (h), a post-nuclear brain fraction (pns), and a membrane-enriched fraction (me) from wild-type mice were used for immunoprecipitation (IP) with mouse or rabbit antibodies against L1 (α-L1) or control IgG. Input and IP samples were subjected to Western blot (WB) analysis with antibodies to ubiquitin and L1 (goat, C-20) (**A**), ATG12 and L1 (mouse, C-2) (**B**), and p62 and L1 (mouse, C-2) (**C**). Arrows indicate the presence of full-length L1 (L1 fl) and of the 70 kDa L1 fragment (L1-70), or of p62, ATG12, and ATG12 in complex with ATG8. Arrowheads indicate bands corresponding to the light and heavy antibody chains and proteins bands that could not be identified.

To investigate whether not only full-length L1 [69,70] but also L1 fragments are ubiquitinated and whether L1 interacts with p62 via ubiquitin, L1 was immunoprecipitated. Brain homogenate and nuclear-enriched and membrane-enriched fractions were incubated with the antibody against the intracellular domain of L1 to precipitate L1 and immunoprecipitated proteins were analyzed with antibodies against ubiquitin and against L1. In contrast to mouse IgG, which was used as control, the L1 antibody precipitated not only L1 but also three to five proteins which were recognized by the ubiquitin antibody (Figure 6A). A strong band of approximately 70 kDa was recognized by the ubiquitin antibody and the L1 antibody. A weak band of approximately 200 kDa was also detected by these antibodies, showing that L1 can be ubiquitinated and that not only full-length L1 but also L1-70 carries ubiquitin (Figure 6A). Furthermore, when analyzing proteins co-immunoprecipitated with L1, we found that L1 co-immunoprecipitates with ATG12 (Figure 6B) and p62 (Figure 6C). ATG12 was co-immunoprecipitated with full-length L1 and the 70 kDa L1 fragment (L1-70) from brain homogenate, and ATG12 in complex with ATG8 was co-immunoprecipitated with full-length L1 and L1-70 from the membrane-enriched fraction, whereas naïve control IgG did not precipitate ATG12 (Figure 6B). p62 was precipitated from brain homogenate with the L1 antibody but not with control IgG, whereas using a membrane-enriched fraction, p62 did not co-immunoprecipitate with L1 (Figure 6C). These results strengthen the view that L1 interacts with ATG12 and p62 not only in biochemical assays but also in a cellular context.

### 2.3. The Interaction of L1 with ATG Family Proteins Is Required for L1-Mediated Amelioration of Aβ-Induced Toxicity

**Figure 7:**
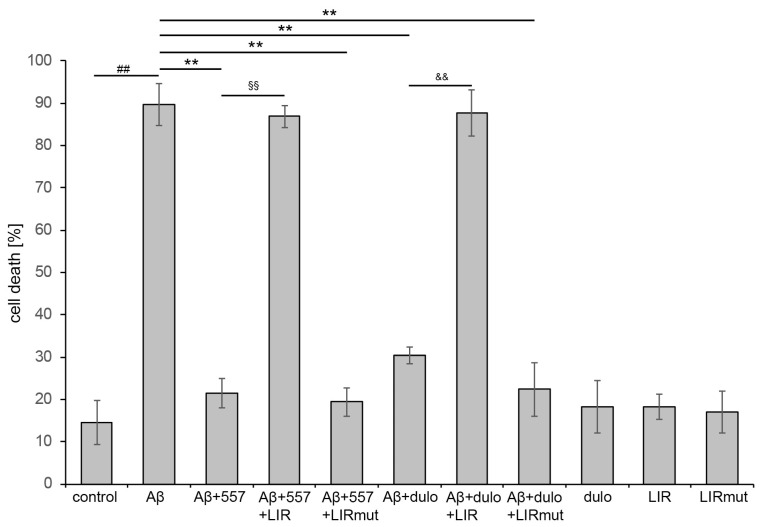
**Amelioration of Aβ-induced toxicity by L1 depends on its interaction with ATG family proteins.** Cortical neurons were allowed to settle down, treated with H_2_O (control), cell-penetrating L1-LIR peptide (LIR), or mutated LIR peptide (LIRmut), cultured overnight, and treated with H_2_O, 50 µg/mL 557 antibody (557), or 100 nM duloxetine (dulo) triggering L1’s functions or 5 µM Aβ peptide for 24 h. Cell death was determined by staining and counting of live and dead cells from 15 fields of three wells per condition and experiment. Mean values ± SD from three independent experiments are shown. Data were analyzed with one-way ANOVA with Bonferroni’s multiple comparison test, ## *p* < 0.01 relative to control treatment, ** *p* < 0.01 relative to Aβ treatment, ^§§^ *p* < 0.01 compared to Aβ + 557 treatment, and ^&&^ *p* < 0.01 compared to Aβ + duloxetine treatment.

In previous studies, we had found that L1 binds to Aβ and that overexpression of full-length L1 or L1-70 in mouse models for Alzheimer’s disease reduces Aβ42 levels, Aβ plaques, and synapse loss [26,41]. Since L1 enhances cell survival under oxidative stress, we determined if L1-mediated cell survival is abolished in the presence of the LIR peptide and if triggering of L1’s functions ameliorates Aβ-induced toxicity. We treated cortical neurons with Aβ, 557 antibody, duloxetine, LIR peptide, and mutated LIR peptide and determined the number of live and dead cells. Aβ-induced toxicity was strongly reduced when cells were treated with L1-function triggering antibody 557 or duloxetine. In the presence of the cell-penetrating LIR peptide, this effect was abolished while the mutated LIR peptide did not reduce the L1-mediated cell survival (Figure 7). The peptides alone did not affect the cell survival, demonstrating that L1-mediated cell survival depends on the interactions of L1 with ATG family proteins like LC3 and ATG12 which are mediated by L1’s LIR domain. Furthermore, these results indicate that triggering of L1’s functions by antibody 557 or duloxetine not only ameliorates Aβ-induced toxicity but also enhances autophagy and clearance of Aβ.

### 2.4. Triggering L1’s Functions Enhances Autophagy of Aβ

**Figure 8:**
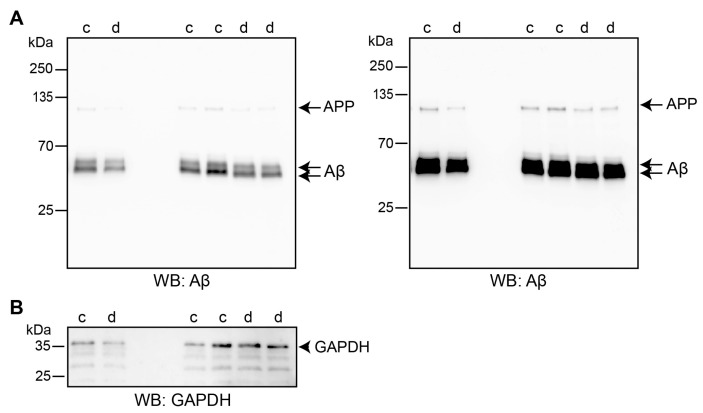
**Duloxetine treatment reduces Aβ levels.** B103 cells were treated with solvent control (c) or 100 nM duloxetine (d) for 48 h; thereafter, cells were lysed and cell lysates from three independent experiments were subjected to Western blot (WB) analysis with rabbit Aβ antibody (short exposure: left image; long exposure: right image) (**A**) followed by an antibody against glyceraldehyde-3-phosphate dehydrogenase (GAPDH; (**B**)). Full blots are shown for the detection of proteins with the Aβ antibody, and the region of interest is shown for the re-probed blot with the GAPDH antibody. The arrows indicate APP and Aβ, and the arrowhead indicates GAPDH.

To substantiate the notion that triggering of L1’s functions by duloxetine could enhance autophagy of Aβ, we investigated co-localization of Aβ with ATG12, p62 and LC3 and determined cellular Aβ levels. B103 cells were treated with duloxetine and proximity ligation was performed using antibodies against Aβ and ATG12. Quantification of the red spots indicating the close proximity of Aβ with ATG12 showed that duloxetine treatment enhanced the number of red spots per cell by a factor of 3.5 (Supplementary Figure S7A,B). Furthermore, in control cells, no co-staining for Aβ and p62 was observed while some co-staining was observable after duloxetine treatment (Supplementary Figure S7C,D,G). No co-staining was found for Aβ and LC3, neither in the control nor duloxetine-treated cells (Supplementary Figure S7E–G), which is in agreement with the previous finding that Aβ aggregates are not incorporated into LC3-positive autophagosomes [68]. We next determined if duloxetine treatment alters overall Aβ levels. For this aim, Western blot analysis was performed. B103 cells were treated with solvent control or duloxetine, and Aβ levels were determined. Levels of Aβ were lower in duloxetine-treated cells compared to levels in control cells (Figure 8). These results suggest that triggering L1’s functions with duloxetine leads to improved cell survival, enhanced autophagy of Aβ, and reduced Aβ levels.

## 3. Discussion

The LIR motif targets autophagy receptors to ATG8 family proteins like LC3, which are anchored to the membrane of the phagophore [55,62]. LIR-containing proteins comprise cargo receptors, members of the basal autophagy apparatus, proteins associated with vesicles and vesicle transport, ‘Ras-related in brain’ (Rab) GTPase activating proteins, and signaling proteins that are degraded by selective autophagy [54]. For normal function and survival of neurons, proper protein synthesis, trafficking, and clearing by autophagy and other processes like endoplasmic reticulum-associated protein degradation are of key importance [71]. Proteostasis failure, aberrant accumulation of proteins, mitochondrial dysfunction, and reactive oxygen species (ROS) inducing oxidative stress are common in neurodegenerative diseases and senescence-related diseases like AD, Parkison’s disease, amyotrophic lateral sclerosis, and age-related macular degeneration and strongly contribute to disease progression and severity [46,59,71,72,73,74,75]. During early stages of AD, protein and lipid oxidation in brain regions rich in Aβ were found [72]. In the presence of metal ions, Aβ peptides produce ROS [74,76]. Furthermore, defects in autophagy and proteostasis are intricately linked to neurodegeneration and AD [49]. Recent studies show that proteins with an LIR motif can protect neurons and AD model mice from tau- and Aβ-induced mitochondrial dysfunction, synapse loss, and decline in cognitive function [77,78]. Disrupted-in-schizophrenia-1 (DISC1) interacts with LC3, a by canonical LIR motif, and is associated with psychiatric disorders and AD. DISC-1 can rescue neurons from Aβ-induced mitochondrial dysfunction, spine loss, and impaired long-term potentiation [77]. Sequestosome-1 (SQSTM1), also called ubiquitin-binding protein p62 (SQSTM1/p62), also called p62, contains an LIR domain and directly interacts with ATG8 and LC3 [79]. p62 rectifies neurofibrillary tangle pathology and spreading of misfolded tau by selective targeting of tau [78]. In addition, the LIR domain containing vacuolar protein sorting ortholog 35 is important for microglial uptake of Aβ, and AD model mice deficient in this protein exhibit decreased learning and memory and increased Aβ plaque load in dystrophic neurites and in reactive astrocytes [80]. Due to the importance of autophagy and proteostasis mechanisms for preserving cellular homeostasis and protein quality control in the aging organism, understanding and controlling autophagic clearance mechanisms and protecting cells against ROS and mitochondrial damage is of key importance. Senescence-related and neurodegenerative diseases are an increasing problem for society [81,82,83]. Thus, unraveling and controlling autophagy and proteostasis will help to develop new treatments of these medical conditions [49,84].

We had identified two LIR motifs in L1, one in its intracellular domain and one in its extracellular domain. The LIR motif in L1’s extracellular fourth FNIII homologous repeat mediates the binding to LC3. Treatment of neurons with a cell-penetrating peptide containing L1’s LIR domain, which disrupts the interaction of L1 with LC3, reduces outgrowth and survival of cultured primary neurons [52]. Furthermore, L1 regulates mitochondrial metabolism and trafficking [30,39]. In addition, L1 interacts with Aβ, reduces Aβ plaque load, and ameliorates the Aβ pathology in AD model mice [26,41,85]. Therefore, L1 is an interesting candidate that could control autophagic clearance, protect cells by reducing ROS levels, and maintain mitochondrial function. To obtain further insights into the neuroprotective function of L1 and its ability to ameliorate the symptoms and progression of neurodegenerative diseases, we here investigated if L1 also interacts with other LIR receptors of the ATG family proteins and how the interaction of L1 with ATG proteins influences autophagy, especially, targeting of Aβ to the autophagosome. We found that L1 interacts with autophagosome-localized protein ATG12 and that interactions of L1 with LC3 and Aβ are enhanced under oxidative stress, whereas the interactions of L1 with ATG12 are not altered by ROS. In addition, triggering L1’s functions with antibody 557 and the small molecule L1 agonist duloxetine enhances the interaction of L1 with ATG12 and this increase is abolished by treatment with the LIR peptide.

ATG12 and LC3 control autophagosome formation, and ATG12 is part of the core autophagy machinery common to all autophagy pathways [54,84], while LC3 is involved in the elongation process of the phagophore [84]. Interestingly, ATG12 also plays a role in the regulation of mitochondrial biogenesis, cellular respiration, energy metabolism, and cell death [86,87]. Our findings here and in our previous study show that a peptide containing the LIR motif in the fourth FNIII homologous repeat not only disrupts the interactions of L1 with LC3 and ATG12 but also reduces L1-mediated cell survival upon hydrogen peroxide treatment and reduces neurite outgrowth of cultured cerebellar and cortical neurons [52] and abolishes L1-mediated survival of cortical neurons treated with Aβ peptide. This underscores the importance of the L1–LC3 and L1–ATG12 interactions for cellular vitality. Since L1 interacts with ATG12 and LC3, it could control simultaneously the two protein complexes that regulate autophagosome formation. We propose that in the first incident, ATG7, ATG5, and ATG12 interact and thereafter associate with ATG16 to elongate the phagophore. Second, LC3 is cleaved by ATG4B and then coupled to the autophagosome by phosphatidylethanolamine (LC3II) [49,71]. Autophagosome-coupled LC3II binds to the adapter protein p62, which is involved in recognition and targeting of ubiquitinated protein aggregates to degradation by selective autophagy.

We now found that not only full-length L1 [69,70], but also the intracellularly localized L1-70 is ubiquitinated. We also found that L1 interacts with p62 in cortical neurons. Stimulating L1 with antibody 557 or duloxetine enhances cell survival and binding of L1 to p62. This stimulation also enhances co-localization of Aβ and p62. Furthermore, the interaction of L1 with p62 is also enhanced in stressed cells, suggesting enhanced targeting of L1 to the autophagosome, thereby enhancing autophagy. Ubiquitination and autophagy of L1 and other proteins are important for controlling the interaction of proteins with ligands and for degradation of misfolded and aggregated proteins [84,88]. Protein aggregates of Aβ and tau are predominantly bound to p62 for degradation [68]. Also, disrupted p62-mediated mitophagy affects the pathogenesis of Parkinson’s disease [89]. Since L1 enhances the co-localization of Aβ and p62, we propose that triggering L1 influences the degradation of protein aggregates of Aβ and tau. Notably, accumulation of defective ubiquitin was detected at early stages of AD in human patients, and expression of defective ubiquitin leads to accumulation of APP and Aβ in a cell culture model [90]. Reduced levels of p62 in the frontal cortex of AD patients and in AD model mice were suggested to be responsible for a decreased nuclear respiratory factor 2-dependent antioxidant response that leads to impaired oxidative stress resistance, thereby contributing to AD pathology [89]. Together with the finding of increased induction of autophagy but impaired autolysosome formation in AD [91], these recent studies reinforce the importance of ubiquitination and p62 function for protein degradation and cellular health. In addition to being a classical autophagy receptor, p62 also plays important roles in cell metabolism, signaling, and apoptosis [89,92]. Also, tumor initiation and progression depend on p62 [93]. Since L1 also plays a role in tumor progression [9,94], it is possible that L1 and p62 interactions are not only important for autophagy but also for tumor progression.

L1’s interactions with LC3, ATG12, and p62 could influence protein degradation by affecting the elongation phase of the autophagosome via ATG12 and LC3 and by directing via p62 ubiquitinated proteins to proteasomes or autophagosomes. Interestingly, p62 not only localizes in the cytosol but also in the nucleus, where it sequesters nuclear proteins during stress [95]. Since L1 fragments, such as L1-70, are present in the nucleus and influence neuronal survival by interacting with nuclear proteins [31,37,38], L1 might also influence nuclear cargo uptake of p62.

Arrest in autophagic degradation of protein aggregates and accumulations of p62 and LC3II are suggested to be upstream events in neurodegenerative diseases, such as Alzheimer’s disease [68]. Furthermore, several studies describe autophagy dysfunction in Alzheimer’s patients and Alzheimer’s disease model mice, and accumulation of autophagic vesicles was suggested to be caused by a dysfunction during autophagosome maturation [96,97,98,99]. In addition, Aβ interferes with retrograde transport of autophagosomes in axons, and hyperphosphorylated tau interferes with autophagosome–lysosome fusion, thereby contributing to pathogenesis and loss of synapses [97,100]. Since altered proteolytic cleavage of L1 was observed in patients with Alzheimer’s disease and other dementias [24], interactions of L1 with LC3, ATG12, and p62 may be altered in these patients. These altered interactions could contribute to disease progression and severity by impairing autophagy and protein degradation. Furthermore, L1 and L1-70 contribute to the clearance of Aβ in AD model mice in vivo [26,41]. This supports the view that L1 affects protein degradation by autophagy. We therefore call L1 an autophagy receptor.

Current therapies for AD, such as treatment with cholinesterase inhibitors (e.g., galantamine, rivastigmine, and donepezil), immunotherapy agents (e.g., lecanemab and donanemab), or memantine, an N-methyl-D-aspartate receptor antagonist, only slow down cognitive decline, thereby reducing some cognitive and behavioral symptoms [101]. Thus, novel therapies to treat the disease are needed. Anti-Aβ monoclonal antibody treatments, vaccines, and natural products that target Aβ oligomers in clinical trials resulted in controversial findings and safety concerns (see, for instance: [101,102,103,104,105]). Since L1 is beneficial for cell functions and enhances autophagy and reduces Aβ plaque load in an AD mouse model it might be a hopeful candidate for AD therapy.

Of note, L1 is not only involved in development, maintenance, and regeneration of the nervous system but also in tumor progression. L1 mediates adhesion and migration of neuronal cells which are necessary for proper nervous system development and regeneration in the adult [2]. L1 is also involved in tumor cell metastasis and often linked to high malignancy of tumors and tumor progression [8,9]. Thus, to develop an L1-mediated therapy for the treatment of neurodegenerative diseases, potential side effects need to be considered. In vivo experiments showed that overexpression of L1 or enhanced generation of L1-70 are beneficial for nervous system regeneration and health and do not lead to unwarranted side effects like tumor induction [26,41,106]. Nonetheless, further experiments are warranted to translate our results into the human context. Also, it will be necessary to compare the mechanisms that enhance tumor cell migration and neural stem cell migration.

Altogether, our findings demonstrate that L1 plays an important role in the degradation of pathologically aggregated proteins via two routes: one depending on ATG members via LC3, the other via ubiquitin. This remarkable function of two parallel mechanisms that aim at cell protection shows that so-called redundancy appears to be an advantage. It will be interesting to investigate whether these two mechanisms influence each other, and if so, whether they control each other in downstream signal transduction not only in Alzheimer’s disease but also in other neurodegenerative diseases and in aging.

## 4. Materials and Methods

### 4.1. Mice

C57BL/6J mice were bred and housed in the animal facility of the Universitätsklinikum Hamburg-Eppendorf at 25 °C on a 12 h light/12 h dark cycle with ad libitum access to food and water. Mice of either sex were used for all experiments. All procedures were conducted in accordance with European Union regulations and with approval of the local authorities of the State of Hamburg (animal permit number ORG_1022). Experimental setup and manuscript preparation were according to the ARRIVE guidelines [107].

### 4.2. Antibodies and Reagents

Mouse monoclonal L1 antibody C-2 (NCAM-L1; sc-514360; no RRID available), goat L1 antibody C-20 (NCAM-L1; sc-1508; no RRID available) recognizing epitopes in the intracellular domain of human and mouse L1, mouse monoclonal ubiquitin antibody recognizing human, mouse, and rat ubiquitin (sc-8017; RRID:AB_628423), mouse monoclonal LC3 antibody recognizing human, mouse, and rat LC3B (sc-376404; RRID:AB_11150489), mouse GAPDH antibody recognizing mouse and human GAPDH (sc-137179; RRID:AB_2232048), and mouse tubulin antibody recognizing alpha-tubulin from human, rat, and mouse (sc-8035; RRID:AB_628408) were from Santa Cruz Biotechnology (Dallas, TX, USA). Function-triggering rat monoclonal L1 antibody 557 against the 3rd fibronectin type III homologous repeat has been described [108]. Rabbit polyclonal antibody against the extracellular domain of murine L1 was generated as described in [109]. Rabbit LC3 antibody recognizing, human, mouse, and rat LC3B (14600-1-AP; RRID:AB_2137737) was from Proteintech Europe (Manchester, United Kingdom). Goat polyclonal antibody against human, mouse, and rat autophagy related protein 12 (ATG12) was from Biozol (BYT-ORB153327; no RRID available; Eching, Germany). Rabbit antibody against human, mouse, and rat Aβ38/40/42/43 (218103; RRID:AB_2056422) and mouse antibody against human, mouse, and rat Aβ38/40/42/43 (218211; RRID:AB_2619916) were from SynapticSystems (Göttingen, Germany). Rabbit p62 antibody recognizing human, rat, and mouse p62/SQSTM1 was from Sigma-Aldrich (P0067-25UL; RRID:AB_1841064; Darmstadt, Germany). Horse-radish peroxidase (HRP)-coupled goat anti-Fc antibody (109-035-190; RRID:AB_2888996) was from Jackson ImmunoResearch (Cambridgeshire, United Kingdom). Fluorescence- and HRP-coupled secondary antibodies and normal rabbit serum and IgG from naïve mice were from Dianova (Hamburg, Germany). (S)-Duloxetine hydrochloride (CAS Number 136434-34-9) was from Tocris Bioscience (Wiesbaden-Nordenstadt, Germany). Human Aβ1-42 peptide with the sequence H-DAEFRHDSGYEVHHQKLVFFAEDVGSNKGAIIGLMVGGVV-OH, mouse L1-LIR peptide containing the extracellular LC3-interacting region (LIR) motif of L1 (_952_LSYHPV_957_) H-SHNGVLTGYLLSYHPVEGESKEQ-OH, the peptide containing the mutated mouse L1-LIR motif SHNGVLTGYLLSAHPAEGESKEQ, mouse L1-LIR peptide containing the cell-penetrating HIV tat-sequence (YGRKKRRQRRR; [110]) YGRKKRRQRRRSHNGVLTGYLLSYHPVEGESKEQ, and the mutated L1-LIR peptide with tat-sequence YGRKKRRQRRRSHNGVLTGYLLSAHPAEGESKEQ were purchased from Schafer-N (Copenhagen, Denmark). Production and purification of recombinant L1Fc containing the extracellular L1 domains of mouse L1 (L1CAM_MOUSE, P11627; amino acids 35-1112) and CHL1Fc containing the extracellular domain of CHL1 (NCHL1_MOUSE, P70232; amino acids 35-1015) fused to Fc from human IgG have been described in [111]. Recombinant human LC3B (UBI-60-0112-500) and recombinant human ATG12 (PRS-95-125; lot 95-125-β9β4) were from BIOZOL (Eching, Germany). 3-Methyladenine (3-MA; sc-205596) was from Santa Cruz Biotechnology, and rapamycin (R0395-1MG) was from Sigma-Aldrich. Monodansylcadaverine (MDC, sc-214851) was from Santa Cruz Biotechnology. Rat B103 neuroblastoma cells (RRID:CVCL_D538) expressing human amyloid precursor protein (APP) with Swedish mutation and HEK293 cells (RRID:CVCL_0045) expressing human APP with Swedish mutation and overexpressing presenilin 1 were a kind gift of Stefan Kins (Rheinland-Pfälzische Technische Universität Kaiserslautern-Landau, Kaiserslauten, Germany) and Ulrike Müller (Institute for Pharmacy and Molecular Biotechnology, Functional Genomics, University of Heidelberg, Heidelberg, Germany). FuGENE^®^ HD Transfection Reagent (E2311) was purchased from Promega (Walldorf, Germany). The plasmid containing human L1 cDNA was a kind gift of Michael K.E. Schäfer (Department of Anesthesiology, University Medical Center of the Johannes Gutenberg-University Mainz, Mainz, Germany) [69].

### 4.3. Isolation and Maintenance of Cortical Neurons and B103 and HEK293 Cells

To obtain dissociated cortical neurons, cerebral cortices from 15.5- to 16.5-day-old wild-type mouse embryos were dissected out, cut into several pieces, and incubated in HBSS containing 0.025% trypsin (Sigma-Aldrich, Darmstadt, Germany) at 37 °C for 30 min. To stop the enzymatic reaction, HBSS containing 1% BSA (Sigma-Aldrich) and 1% w/v trypsin inhibitor (T-6522, Sigma-Aldrich) was added to the tissue for 5 min at 37 °C. After washing in HBSS, the tissue was mechanically dissociated. The dissociated cells were maintained in Neurobasal medium (ThermoFisher Scientific, Darmstadt, Germany) containing 1% B27, 2 mM L-glutamine, 100 U/mL penicillin, and 100 μg/mL streptomycin. For the proximity ligation assay, immunohistochemistry, and determination of autophagy, cells were seeded onto poly-L-lysine-coated 12 mm coverslips in a 24-well plate at a density of 2.5 × 10^5^ cells per well or onto poly-L-lysine-coated 6-wells at a density of 2 × 10^6^ cells per well. For triggering L1 functions, neurons were treated with 50 µg/mL L1 antibody 557 for 2 h or 100 nM duloxetine for 24 h or 48 h at 37 °C. To disturb the interaction of L1 with LC3B and ATG12, cells were treated with the LIR peptide from the extracellular domain of L1 or with a mutated LIR peptide at 100 µg/mL 30 min after seeding. For experiments with autophagy inhibitors or to induce oxidative stress, neurons were treated 16 h after seeding with 100 µM 3-methyladenine, 100 nM rapamycin, or 10 µM hydrogen peroxide for 24 h. To trigger L1, cells were treated with monoclonal L1 antibody 557 at 25 µg/mL or S-duloxetine hydrochloride at 100 nM for 24 or 48 h. To determine neuronal survival, cortical neurons were seeded at a density 1.25 × 10^5^ cells per well of a 48-well plate coated with poly-L-lysine. Neurons were maintained overnight in the serum-free medium and then treated with cell-penetrating LIR or LIRmut peptides with tat-sequence (50 µg/mL), L1 antibody 557 (50 µg/mL), and 0.001% DMSO (solvent control for duloxetine) or duloxetine (100 nM) and exposed to Aβ-mediated toxicity by the addition of 5 µM Aβ1-42 peptide for 24 h. Cells were then stained with calcein-AM (Thermo Fisher Scientific) and propidium iodide (Sigma-Aldrich) for 30 min at 37 °C to mark live and dead cells and then directly imaged with a Zeiss AxioObserver.A1 micro-scope (Carl Zeiss, Jena, Germany) with a 20× objective (aperture 0.4) and the AxioVision 4.6 software (Carl Zeiss). Live and dead cells were counted in five images (containing 350–400 cells each) from each of three wells per condition and experiment using ImageJ (ImageJ, version 2.15.0; RRID:SCR_003070; https://imagej.net/; RRID:SCR_003070, accessed on 28 January 2024) [112].

B103 rat neuroblastoma cells expressing APP with Swedish mutation and expressing L1 and HEK293 cells expressing APP with Swedish mutation and overexpressing presenilin 1 but not expressing L1 were maintained in high-glucose Dulbecco’s Modified Eagle Medium (DMEM) containing 2 mM L-glutamine, 10% fetal calf serum, and 2% penicillin/streptomycin. Cells were passaged every three to four days and cultured at 37 °C and 5% CO_2_. For transient transfection, HEK293 cells were seeded at a density of 2 × 10^5^ cells/mL on poly-L-lysine-coated glass coverslips and maintained until they reached a density of 60–70%. Then, the medium was exchanged with DMEM/FCS without antibiotics, and 1 µg plasmid containing human L1 cDNA or an empty plasmid and 3 µL FuGENE transfection reagent in 100 µL DMEM were added. After 6 h, the medium was replaced with DMEM/FCS containing penicillin/streptomycin, and cells were cultured for additional 18 h before treatment. B103 cells were seeded at a density of 2 × 10^5^ cells/mL on poly-L-lysine-coated glass coverslips and maintained for 48 h in DMEM/FCS before treatment. Transfected HEK293 cells and B103 cells were treated with H_2_O (control) or solvent control (0.1% DMSO stock; 1:100), 20 µg/mL H_2_O_2_, 100 µM 3-methyladenine, 100 nM rapamycin, 100 µg/mL LIR peptide, 100 µg/mL mutated peptide, 50 µg/mL 557 antibody, or 100 nM duloxetine (10 µM stock in 0.1% DMSO) for 24 h or 48 h. For Western blot analysis, cells were seeded at a density of 1 × 10^6^ cells/well into 6-wells and cultured for 48–96 h. Cells were lysed with 200 µL lysis buffer (50 mM Tris/HCl (pH 7.5), 150 mM NaCl, 1% NP40, 1 mM Na4P2O4, 1 mM NaF, 1 mM EDTA, 2 mM Na_3_VO_4_, and 1 mM PMSF) pre 6-well and cell lysates were mixed with SDS-sample buffer (60 mM Tris/HCl, pH 6.8, 2% SDS, 1% β-mercaptoethanol, 10% glycerol, 0.02% bromophenol blue) and boiled for 5 min before use.

### 4.4. Enzyme-Linked Immunosorbent Assay (ELISA)

For ELISA, 384-well microtiter plates with high binding surface (Corning, Tewksbury, MA, USA) were incubated with 25 µL of 10 µg/mL recombinant human LC3B or ATG12 overnight at 4 °C. Wells were washed 5 times for 1 min with Dulbecco’s phosphate-buffered saline solution containing MgCl_2_ and CaCl_2_ (Sigma-Aldrich; D8662) (PBS) and treated with blocking solution (2% essentially fatty acid-free bovine serum albumin in PBS) for 2  h at room temperature. Wells were washed 5× for 1 min with PBS containing 0.005% Tween 20 (PBST). As ligand, the extracellular domain of L1 fused to the Fc part of human IgG was used to present not monomeric domains to the ligand but dimers that bind more effectively. This soluble L1Fc chimera was also shown to act in a similar manner as cell-associated L1 [113]. Wells were incubated with 25 µL of 1–200 µg/mL L1Fc as ligand in the absence and presence of a 20-fold molar excess of Aβ, L1-LIR peptide, or mutated LIR peptide as a competitor for 1 h at room temperature under gentle agitation. As negative control, wells were incubated with CHL1Fc or Fc. After washing 2× with PBS and 3× with PBST for 1 min, HRP-coupled anti-Fc antibody (1:2000) in blocking solution was applied for 1 h at room temperature. Next, samples were washed 2× with PBS and 3× with PBST for 1 min. To detect bound proteins, wells were incubated with 1 mg/mL ortho-phenylenediamine dihydrochloride (ThermoFisher Scientific) for 2–5 min at room temperature in the dark. The enzymatic reaction was stopped by adding 25 µL 2.5 M sulphuric acid to the wells. The absorbance of the samples was determined at 492 nm with an ELISA reader (µQuant; BioTek, Bad Friedrichshall, Germany).

### 4.5. Proximity Ligation Assay

For fixation, cells were incubated for 30 min at room temperature in 4% formaldehyde in PBS. After washing 3× with PBS, cells were subjected to proximity ligation assay (PLA) using Duolink PLA products according to the manufacturer’s protocol (Sigma-Aldrich; Duolink PLA technology) with minor modifications. Cells were permeabilized with PBS supplemented with 1% Triton X-100 for 30 min at room temperature, blocked for 1 h at 37 °C using Duolink Blocking solution, and incubated for 24 h at 4 °C with L1 antibody C-2, LC3 antibody, mouse or rabbit Aβ antibody, ATG12 antibody, or p62 antibody (diluted 1:15 (L1 antibody), 1:20 (LC3, p62 and ATG12 antibodies) and 1:100 (Aβ antibodies) in Duolink Antibody Diluent). Cells were washed 2× for 5 min with Duolink Wash Buffer A at room temperature and incubated for 1 h at 37 °C with a mixture of secondary antibodies conjugated with oligonucleotides (Duolink Anti-Mouse PLA Probe MINUS and Duolink Anti-Rabbit PLA Probe PLUS or Duolink Anti-Goat PLA Probe PLUS, diluted in Duolink antibody diluent according to the manufacturer’s protocol). The following proximity ligation reaction, amplification reaction, and washing steps with Duolink Wash Buffers A and B were then performed according to the manufacturer’s protocol using the Duolink In Situ Detection Reagent RED. Thereafter, the coverslips were mounted in RotiMount containing DAPI (Carl Roth, Karlsruhe, Germany). For each experiment, ten images per condition (5 images per coverslip; 2 coverslips per condition) were taken using an Olympus F1000 confocal microscope (Evident Europe GmbH, Hamburg, Germany) with 60× magnification. Images from three independent experiments were analyzed using the ImageJ software (version 2.15.0).

### 4.6. Immunocytochemistry

Cells on glass coverslips were fixed for 30 min at room temperature in 4% formaldehyde in PBS, washed 3× 1 min with PBS, blocked, and permeabilized with PBS containing 2% bovine serum albumin and 0.5% Triton X-100 for 1 h at room temperature. After one wash with PBS for 1 min, cells were incubated with Aβ (1:500), L1 (1:25), LC3 (1:50), ATG12 (1:100), and p62 (1:100) antibodies overnight at 4 °C. Coverslips were washed 2× for 2 min with PBS, incubated with fluorescence-coupled secondary antibodies (diluted 1:200 in PBS) for 1 h at room temperature, washed again 2× for 2 min with PBS, and mounted in RotiMount (Carl Roth, Karlsruhe, Germany) with our without DAPI. Images were captured at 60× magnification (FV1000, Olympus, Tokyo, Japan). At least 3 areas per coverslip and two coverslips per condition were analyzed. Image analysis was carried out using the ImageJ software (ImageJ; version 2.15.0), and the JACoP plugin [114] was used to calculate the Manders’ correlation values for intensities above Costes threshold and to calculate the Pearson’s correlation coefficient.

### 4.7. Immunoprecipitation and Western Blot Analysis

For immunoprecipitation brain homogenates, a post-nuclear fraction and a membrane-enriched fraction were prepared from three-week-old wild-type mice. Brains were homogenized in homogenization buffer (50 mM Tris/HCl, pH 7.4, 150 mM NaCl, 1% NP-40, protease inhibitor cocktail (Sigma-Aldrich)) and samples were either frozen and stored for immunoprecipitation or centrifuged at 200× *g* and 4 °C for 10 min. The 200 g supernatant was collected and either frozen and stored for immunoprecipitation or centrifuged at 17,000× *g*. The 17,000 g pellet was collected for further analysis. From the crude homogenate, the 200 g supernatant, and the 17,000 g pellet, 500 µg protein were diluted in homogenization buffer and incubated overnight at 4 °C with 1 µg L1 antibody C-2 (SantaCruz Biotechnology), 10 µL rabbit L1 antibody, and 1 µg naïve mouse IgG or 10 µL normal rabbit pre-immune serum (as control IgGs). Afterwards, 20 µL magnetic Protein A or Protein G beads (ThermoFisher Scientific) were added and samples were incubated for 2 h at 4 °C. Beads were then washed trice with homogenization buffer, and 40 µL SDS sample buffer (60 mM Tris/HCl, pH 6.8, 2% SDS, 1% β-mercaptoethanol, 10% glycerol, 0.02% bromophenol blue) was added and beads were boiled for 5 min at 95 °C to elute the proteins bound to the magnetic beads.

For Western blot analysis [28], 100 µg protein from input fractions, 10 µL from IP fractions, or 25 µg protein from cell lysates were applied to the gel and run on 4–20% Mini-PROTEAN^®^ TGXTM Precast Protein Gels or 4–20% CriterionTM TGXTM Precast Midi Protein Gels (BioRad, Feldkirchen, Germany). As a molecular weight marker, PageRuler™ Plus Prestained Protein Ladder (Thermo Fisher Scientific) was applied to the gel. Proteins were transferred to 0.45 µm ProtranTM nitrocellulose membranes (VWR, Darmstadt, Germany); the membranes were washed with Tris-buffered saline solution pH 7.4 (TBS) for 10 min and then incubated for 1 h in blocking solution (5% non-fat dry milk powder in TBS with 0.05% Tween 20 (TBST)) and then incubated overnight with L1 antibody C-2 (1:1000; 0.2 µg/mL), L1 antibody C-20 (1:200), ubiquitin antibody (1:500), ATG12 antibody (1:1000), p62 antibody (1:500), Aβ (1:5000), GAPDH (1:1000), or tubulin antibody (1:1000) in blocking solution at 4 °C with shaking. After washing 5× for 5 min with TBST, membranes were incubated for 1 h at room temperature with HRP-conjugated secondary antibodies (1:20,000 in blocking solution). Chemiluminescent solution (ECL Prime and Select Western blotting reagents; GE Healthcare, Solingen, Germany) and a CCD camera (ImageQuant LAS-4000 mini; GE Healthcare) were used detect the protein bands.

### 4.8. Statistical Analysis

The GraphPad Prism 8 software (RRID:SCR_000306; GraphPad Software, Boston, MA, USA) and SigmaPlot software 14.0 (RRID:SCR_003210; Systat, Palo Alto, CA, USA) were used to conduct the statistical analyses. Statistical tests used for comparisons are specified in the figure legends. With the Shapiro–Wilk test, normal distribution of the data was determined. For data with normal distribution, Student’s *t* test was used to compare two groups. To compare more than two groups, we performed ANOVA followed by a Tukey’s test for multiple comparison. Groups containing low sample numbers and or not normally distributed data were analyzed using the Mann–Whitney U-test for comparing two groups and the Kruskal–Wallis test by ranks followed by Dunn’s multiple comparison test when there were more than two groups. All numerical data are presented as group mean values with standard error of the mean (SEM) or standard deviation (SD).

## Data Availability

Data will be made accessible upon reasonable request.

## Supplementary Materials

The following supporting information can be downloaded at: https://www.mdpi.com/article/10.3390/ijms251910829/s1.
