# Supplementary material for: Functional Relationships between L1CAM, LC3, ATG12, and Aβ
Source: Int J Mol Sci. 2024 Oct 9;25(19):10829. doi: 10.3390/ijms251910829 (PMC11476435; doi:10.3390/ijms251910829)
Supplement: Supplementary file 1 [file ijms-25-10829-s001.zip › ijms-3244244-supplementary.pdf]

Functional relationships between L1CAM, LC3, ATG12 and Aβ

Gabriele Loers, Ute Bork and Melitta Schachner

Supplementary Materials

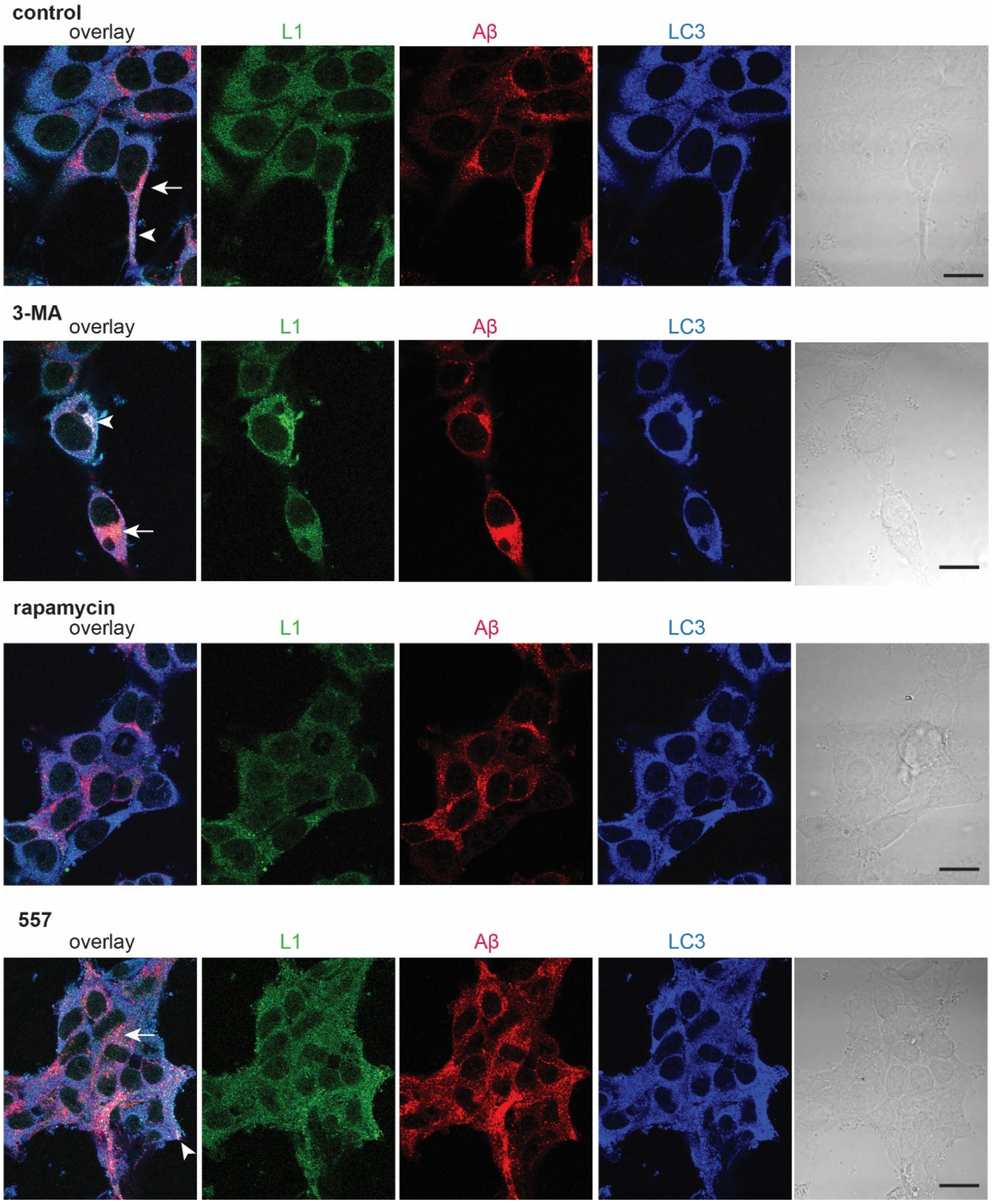

Supplementary Figure S1. Co-localization of L1, Aβ and LC3 in B103 cells is enhanced after treatment with autophagy inhibitor (3-MA). B103 cells were cultured for 24 h, treated with PBS (control) or 50 µg/ml 557 antibody for 4 h, then with 0.1% DMSO (control), 50 µM 3-MA or 100 nM rapamycin for 24 h, fixed and stained with antibodies against L1, Aβ and LC3. Representative images from three independent experiments are shown. Arrows point to yellow dots indicating co-staining of L1 and Aβ. Arrowheads point to white dots indicating co-staining of L1, Aβ and LC3. Scale bars: 20 µm.

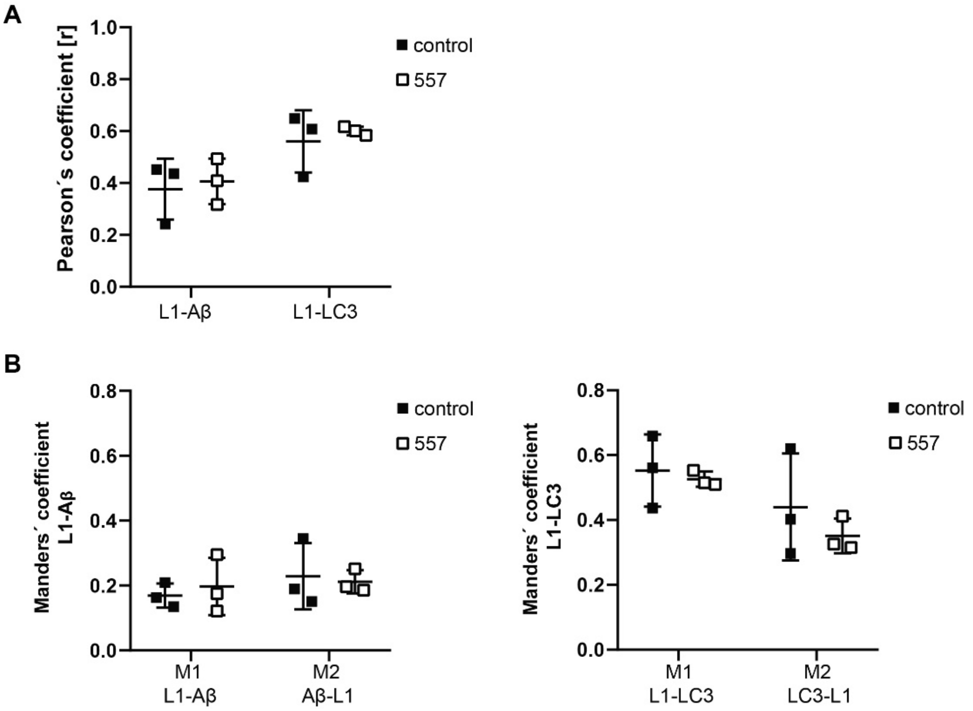

Supplementary Figure S2. L1 co-localizes with Aβ and LC3 in B103 cells. B103 cells were cultured for 48 h, treated with PBS (control) and 50 µg/ml 557 antibody for 4 h, cultured for additional 24 h, fixed and stained with antibodies against L1, Aβ and LC3. Co-localization analyses between L1 (green), Aβ (red) and LC3 (blue) was estimated and the Pearson´s coefficient and the Manders´ co-localization coefficients were determined from three independent cultures. M1: proportion of L1 (green) overlapping with Aβ (red) or LC3 (blue) over the total intensity; M2: proportion Aβ (red) or LC3 (blue) overlapping with L1 (green) over the total intensity. Single values and means with SD from three independent experiments are presented. Data were analyzed with one-way ANOVA with Bonferroni´s multiple comparison test, $p > 0.05$, relative to control treatment.

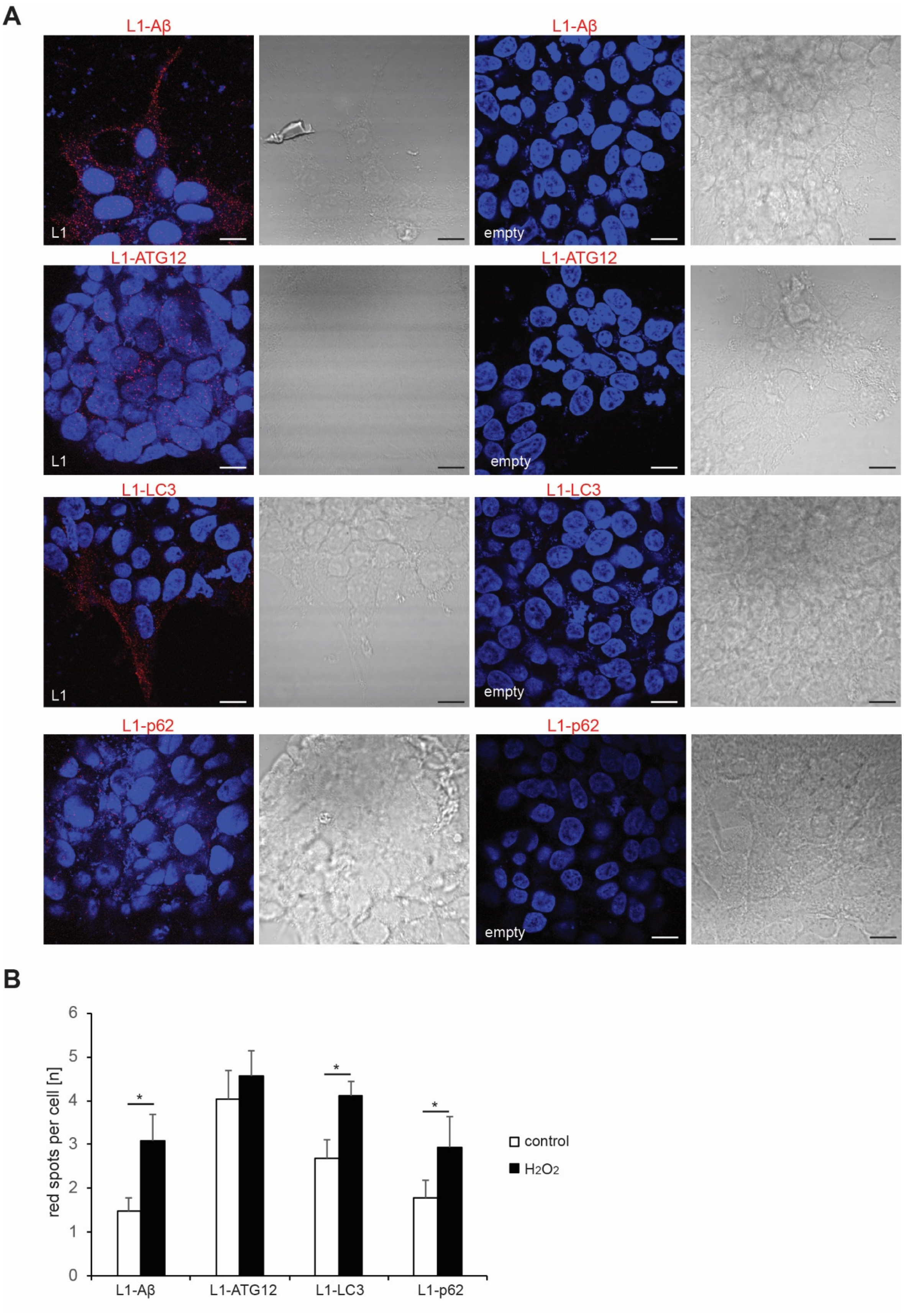

Supplementary Figure S3. Interactions of L1 with Aβ, LC3, ATG12 and p62 in HEK293 cells. HEK293 cells were cultured for 24 h, transfected to express human L1 or transfected with the empty plasmid (empty), thereafter cultured for 24 h and then treated with H_2O (solvent control) or 20 µM H_2O_2 for 24 h. Afterwards, cells were fixed and subjected to proximity

ligation assay with mouse L1 antibody C-2 and rabbit Aβ antibody, rabbit LC3 antibody, goat ATG12 antibody and rabbit p62 antibody. Nuclei were stained with DAPI (blue). (A) Representative images are shown and red spots indicate close interaction of L1 with Aβ, L1 with LC3, L1 with ATG12 and L1 with p62 in cells transfected to express L1, while cells transfected with the empty plasmid only show red spots for Aβ and ATG12. Nuclei were stained with DAPI (blue). Scale bars: 20 µm. (B) Mean values + SD are shown for the average numbers of L1/Aβ-, L1/LC3-, L1/ATG12- and L1/p62-positive red spots per cell from three independent experiments. Data were analyzed with one-way ANOVA with Bonferroni´s multiple comparison test, * $p < 0.01$ relative to control treatment.

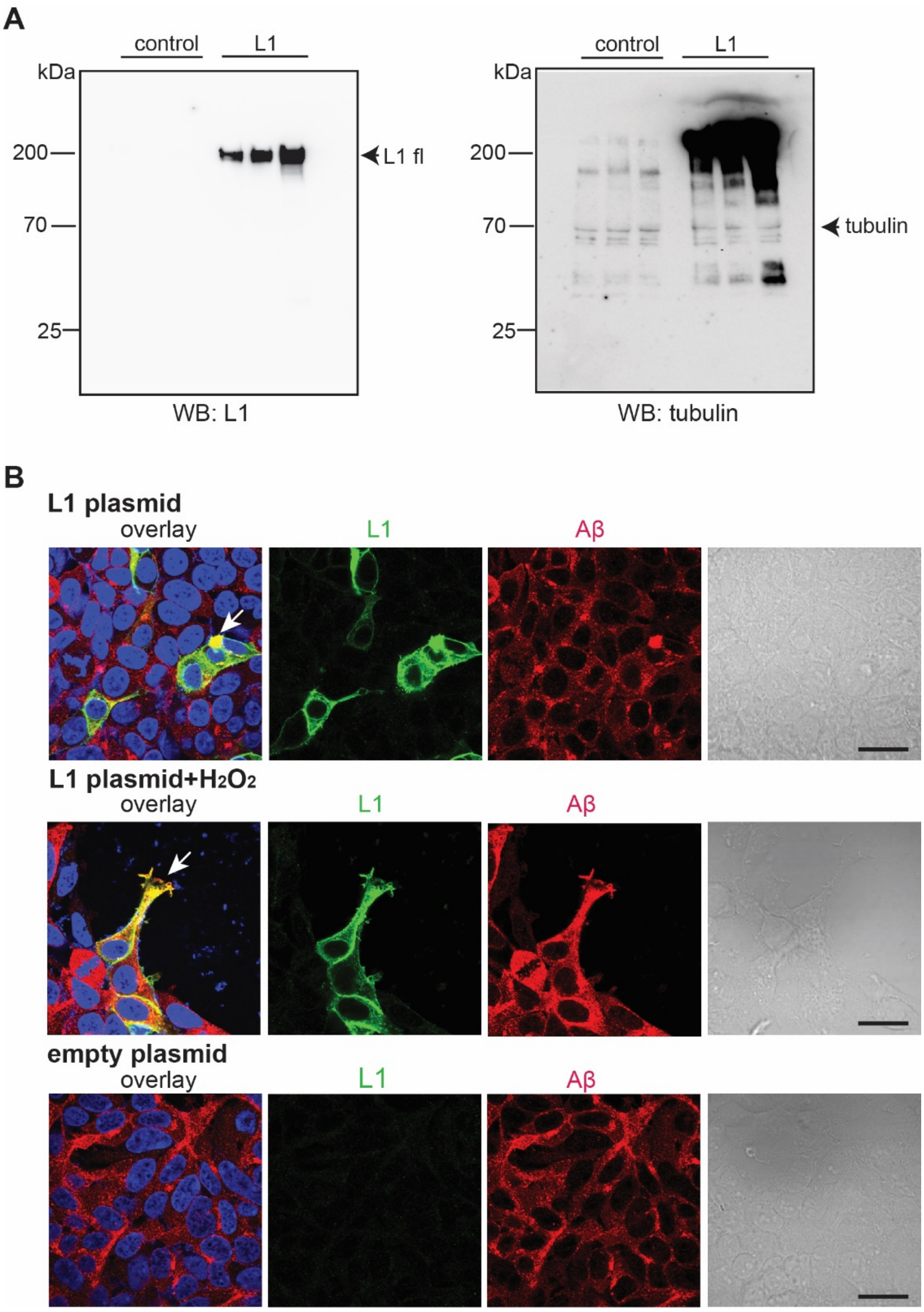

Supplementary Figure S4. L1 expression in transfected HEK293 cells. HEK293 cells were cultured for 24 h, transfected to express human L1 or treated with the empty plasmid, cultured for 24 h, treated with H_2O or 20 µM H_2O_2 for 24 h, fixed or lysed and subjected to Western blotting and immunostaining. (A) Cell lysates from cells transfected with the empty plasmid (control) or L1 plasmid (L1) were subjected to Western blot (WB) analysis with mouse L1 antibody C-2 and re-probed with mouse tubulin antibody to show that proteins were similarly loaded to all lanes. A representative image of a full blot is shown. Arrows point to L1 and tubulin bands. (B) Representative images of cells transfected with the empty plasmid or the L1 plasmid, treated with H_2O or H_2O_2 (+H_2O_2) and stained with mouse L1 antibody C-2 and rabbit Aβ antibody from two independent experiments are shown. Nuclei stained with DAPI (blue) are shown in the overlay. Scale bars: 20 µm. Arrows point to yellow dots showing co-staining of L1 and Aβ.

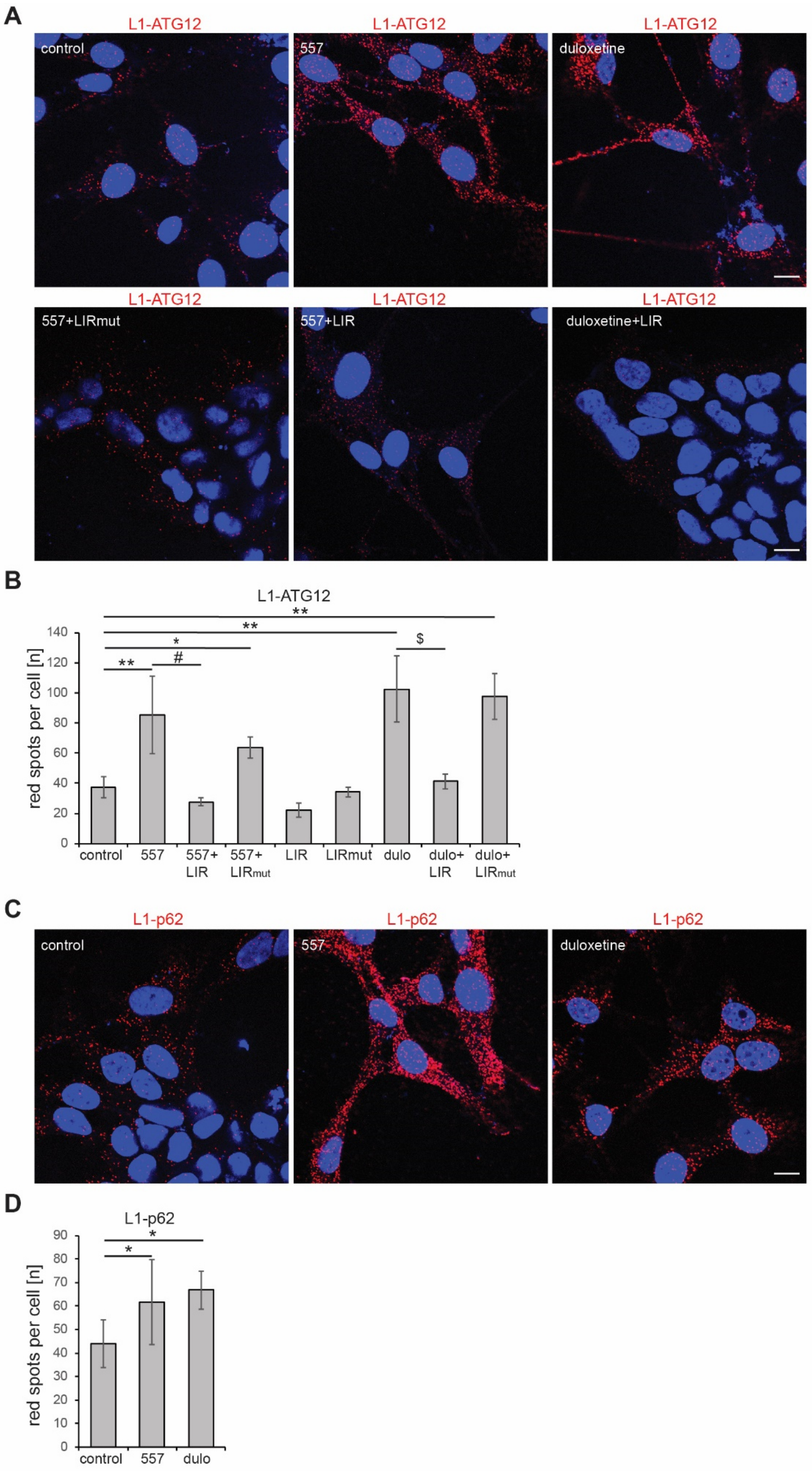

Supplementary Figure S5. Triggering of L1 functions enhances the interactions of L1 with ATG12 and p62. B103 cells (A-D) were allowed to settle down, treated with 0.001% DMSO (solvent control), cell-penetrating L1-LIR peptide or mutated LIR peptide for 30 min, and then

with PBS or function-triggering L1 antibody 557 (557) or L1 agonist duloxetine (dulo) for 24 h. Cells were then fixed and subjected to proximity ligation assay with mouse L1 antibody C-2, goat ATG12 antibody or rabbit p62 antibody. Nuclei were stained with DAPI (blue). (A, C) Representative images are shown and red spots indicate close interaction of L1 with ATG12 (A) and L1 with p62 (C). Scale bars: 20 µm. (B, D) Mean values ± SD are shown for the average numbers of L1/ATG12-positive (B) and L1/p62-positive (D) red spots per cell from three independent experiments. Data were analyzed with one-way ANOVA with Bonferroni´s multiple comparison test, * < 0.05 relative to control treatment, ** $p < 0.01$ relative to control treatment, $^{\#}$ < 0.05 relative to 557 antibody treatment, $^{\$}$ $p < 0.05$ relative to duloxetine treatment.

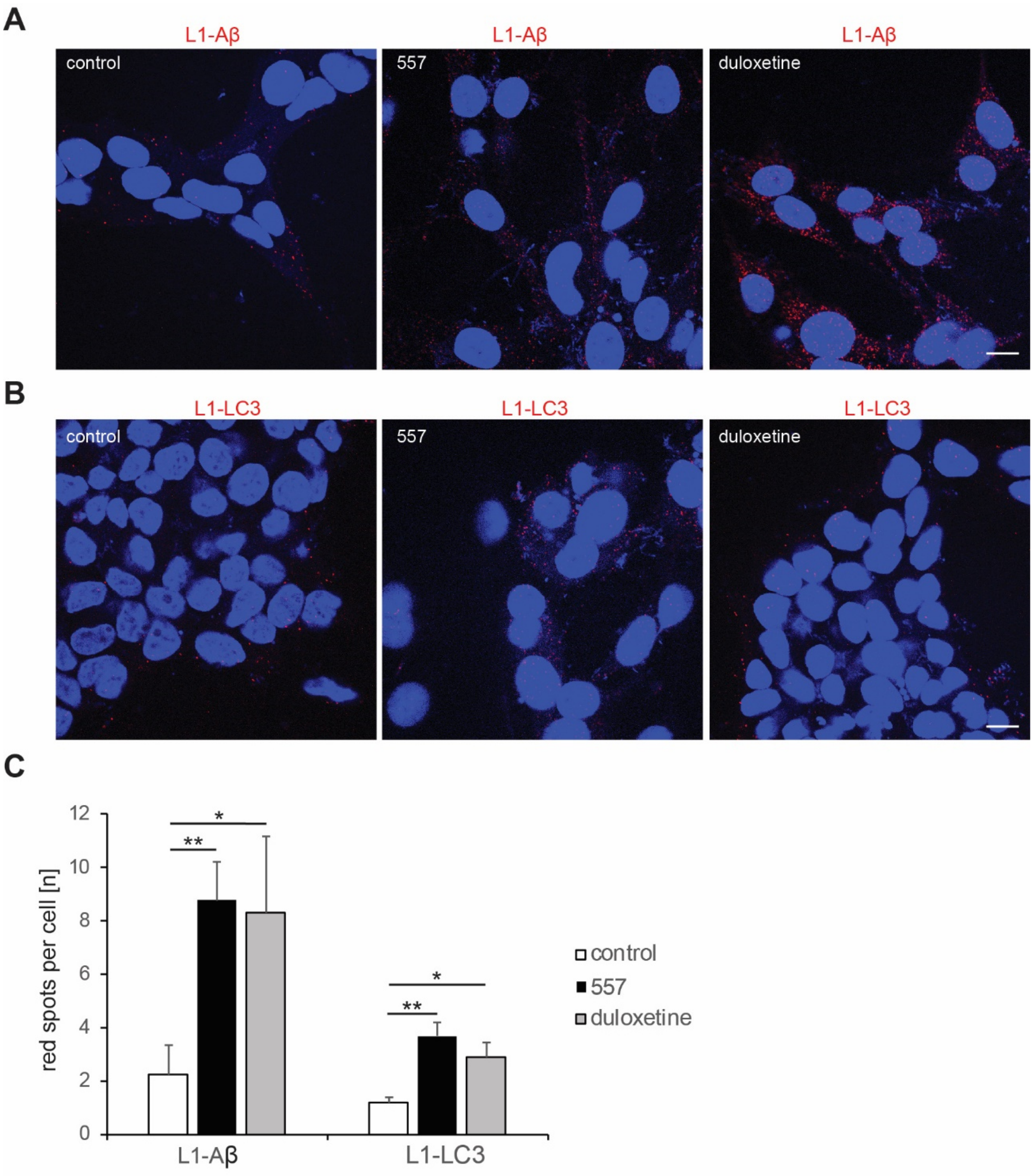

Supplementary Figure S6. Triggering of L1 functions enhances the interactions of L1 with Aβ and LC3. B103 cells (A-C) were allowed to settle down, treated with 0.001% DMSO (solvent control), function-triggering L1 antibody 557 (557) or L1 agonist duloxetine for 24 h. Cells were then fixed and subjected to proximity ligation assay with mouse L1 antibody C-2, rabbit Aβ antibody or rabbit LC3 antibody. Nuclei were stained with DAPI (blue). (A, B) Representative images are shown and red spots indicate close interaction of L1 with Aβ (A) and L1 with LC3 (B). Scale bars: 20 µm. (C) Mean values + SD are shown for the average numbers of L1/Aβ-positive and L1/LC3-positive red spots per cell from three independent experiments. Data were analyzed with one-way ANOVA with Bonferroni´s multiple comparison test; * < 0.05, ** $p < 0.01$ relative to control treatment.

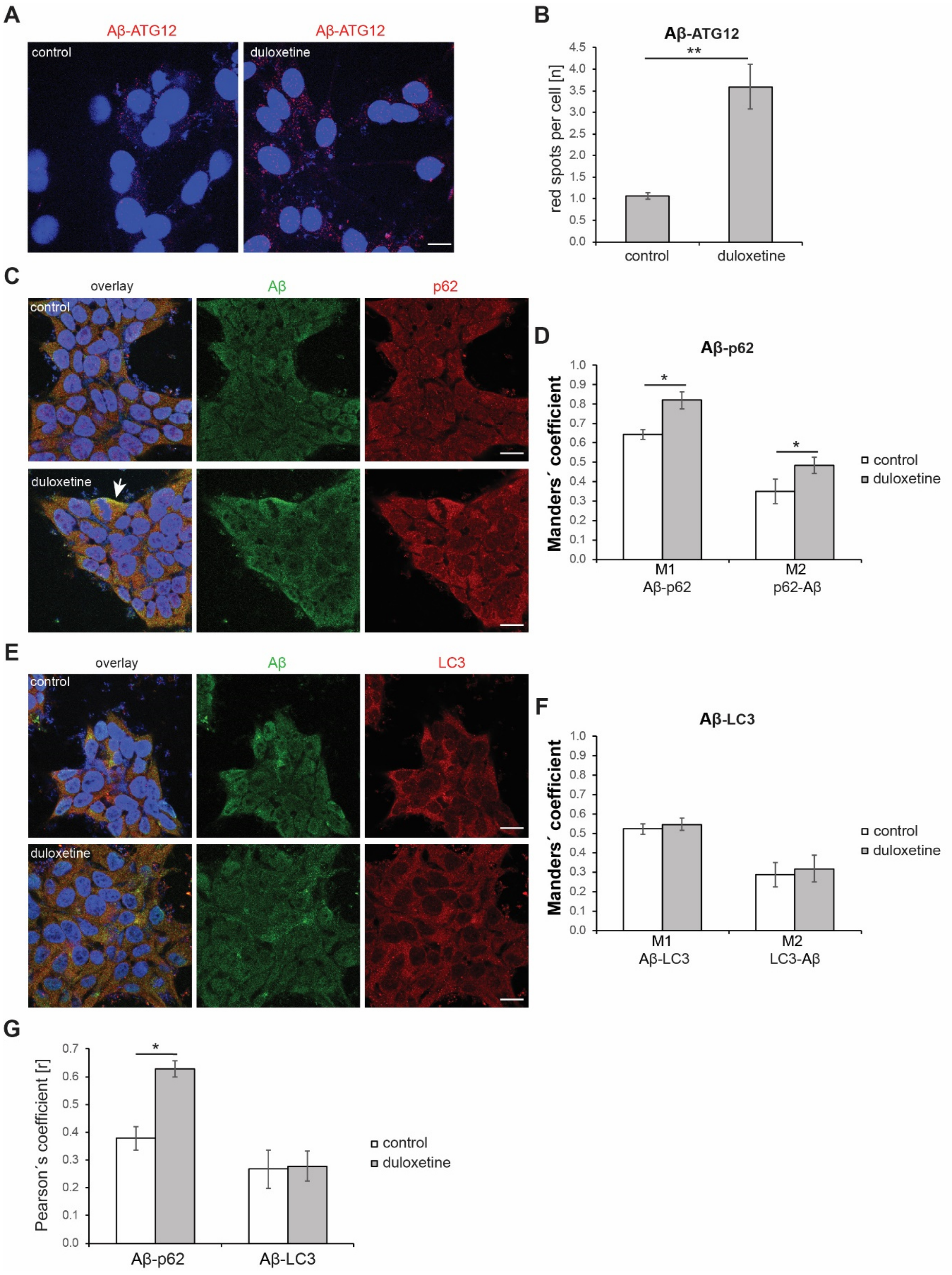

Supplementary Figure S7. Triggering of L1 functions leads to enhanced co-localization of Aβ with ATG12 and Aβ with p62 but not of Aβ and LC3. B103 cells (A-D) were allowed to settle down and treated with 0.001% DMSO (solvent control) or L1 agonist duloxetine for 24 h. Cells were then fixed and subjected to proximity ligation assay with rabbit Aβ antibody and goat ATG12 antibody or immunostaining with rabbit or mouse Aβ antibody, mouse LC3 antibody and rabbit p62 antibody. Nuclei were stained with DAPI (blue). (A) Representative images are shown and red spots indicate close interaction of Aβ with ATG12. Scale bars:

20 µm. (B) Mean values ± SD are shown for the average numbers of Aβ/ATG12-positive red spots per cell from three independent experiments. Data were analyzed with one-way ANOVA with Bonferroni´s multiple comparison test, ** $p < 0.01$, relative to control treatment. (C, E) Representative images are shown for immunostaining of Aβ (green) and p62 (red) (C) and Aβ (green) and LC3 (red) (E). Nuclei were stained with DAPI (blue). Scale bars: 20 µm. (D, F, G) The Manders´ co-localization coefficients (D, F) and the Pearson´s coefficient (G) were determined from three independent cultures. M1: proportion of Aβ (green) overlapping with p62 (red) or LC3 (red) over the total intensity; M2: proportion of p62 (red) or LC3 (red) overlapping with Aβ (green) over the total intensity. Means with SD from three independent experiments are presented. Data were analyzed with one-way ANOVA with Bonferroni´s multiple comparison test, * $p < 0.05$, relative to control treatment.
